# HER2-mediated enhancement of Ebola virus entry

**DOI:** 10.1371/journal.ppat.1008900

**Published:** 2020-10-14

**Authors:** Makoto Kuroda, Peter Halfmann, Yoshihiro Kawaoka

**Affiliations:** 1 Department of Pathobiological Sciences, School of Veterinary Medicine, Influenza Research Institute, University of Wisconsin–Madison, Madison, Wisconsin, United States of America; 2 Department of Microbiology and Immunology, Division of Virology, Institute of Medical Science, University of Tokyo, Tokyo, Japan; 3 Department of Special Pathogens, International Research Center for Infectious Diseases, Institute of Medical Science, University of Tokyo, Tokyo, Japan; Division of Clinical Research, UNITED STATES

## Abstract

Multiple cell surface molecules including TAM receptors (TYRO3, AXL, and MERTK), a family of tyrosine kinase receptors, can serve as attachment receptors for Ebola virus (EBOV) entry into cells. The interaction of these receptors with EBOV particles is believed to trigger the initial internalization events that lead to macropinocytosis. However, the details of how these interactions lead to EBOV internalization have yet to be elucidated. Here, we screened receptor tyrosine kinase (RTK) inhibitors for anti-EBOV activity by using our previously established biologically contained Ebola virus that lacks the VP30 gene (EBOVΔVP30) and identified several RTKs, including human epidermal growth factor receptor 2 (HER2), as potential targets of anti-EBOV inhibitors and as novel host factors that have a role in EBOV infection. Of these identified RTKs, it was only HER2 whose knockdown by siRNAs impaired EBOVΔVP30-induced AKT1 phosphorylation, an event that is required for AKT1 activation and subsequent macropinocytosis. Stable expression of HER2 resulted in constitutive activation of AKT1, resulting in the enhancement of EBOVΔVP30 growth, EBOV GP-mediated entry, and macropinocytosis. Moreover, we found that HER2 interacts with the TAM receptors, and in particular forms a complex with TYRO3 and EBOVΔVP30 particles on the cell surface. Interestingly, HER2 was required for EBOVΔVP30-induced TYRO3 and AKT1 activation, but the other TAM receptors (TYRO3 and MERTK) were not essential for EBOVΔVP30-induced HER2 and AKT1 activation. Our findings demonstrate that HER2 plays an important role in EBOV entry and provide novel insights for the development of therapeutics against the virus.

## Author summary

Ebola virus disease is a global threat to human health as seen during the 2013–2016 outbreak in West Africa and the on-going outbreak in the Democratic Republic of the Congo. Currently, there are no approved therapeutics for patient treatment; although, recent monoclonal antibody therapeutic treatments have been used during the current outbreak, despite their lack of efficacy in the later stage of infection. Here, we screened a library of receptor tyrosine kinase inhibitors for anti-Ebola virus activity and identified five compounds that significantly attenuated Ebola virus growth in cell culture mediated not only by the Ebola virus GP but also by other filovirus GPs. Further investigation focusing on the role of human epidermal growth factor receptor 2 (HER2), the cellular target of two of these inhibitors, in Ebola virus cell entry revealed that HER2 interacts with TAM (TYRO3, AXL, and MERTK) receptors and mediates Ebola virus-induced TYRO3 and AKT1 activation that leads to the initiation of macropinocytic uptake of viral particles into cells. Our findings provide new insights into the mechanism underlying Ebola virus entry, which will be of value to the development of novel therapeutics against the virus.

## Introduction

Ebola virus (EBOV) belongs to the genus *Ebolavirus* in the family *Filoviridae*, which includes four other genera (*Cuevavirus*, *Marburgvirus*, *Striavirus*, and *Thamnovirus*) [1], and causes a severe hemorrhagic fever in humans and non-human primates. Between 2013 and 2016, the largest EBOV outbreak occurred in West Africa and caused over 28,000 cases and more than 11,100 deaths [2]. The second largest outbreak, which occurred in the Democratic Republic of the Congo from 1 August 2018 to 25 June 2020, resulted in 3,317 confirmed cases and 2,287 deaths [3].

EBOV is an enveloped virus with a negative-sense single-stranded RNA genome that encodes at least seven structural proteins: nucleoprotein (NP), viral protein (VP) 35, VP40, glycoprotein (GP), VP30, VP24, and an RNA-dependent RNA polymerase (L). EBOV cell entry, which is one of the targets for therapeutic development, is mediated by the only surface viral protein, GP, which is highly glycosylated. To gain entry into cells, virus particles first attach to the cell surface via interactions with multiple host receptors; for example, C-type lectins that bind to mucin and glycan on GP [4–6], and TIM/TAM phosphatidylserine (PS) receptors that bind to PS on the viral envelope [7–9]. Attached particles are thought to be internalized into early endosome via macropinocytosis due to their large, filamentous structure [10–12]. Virions are then delivered to late endosomes/lysosomes, the mucin domain and glycan cap of GP are removed by cathepsin B/L, and a concomitant conformational change occurs under low pH conditions leading to the exposure of the receptor binding domain (RBD) [13]. The RBD of the processed GP interacts with the endosomal membrane protein Niemann–Pick C1 [14, 15], which triggers fusion of the viral envelope with endosomal membrane, resulting in the release of viral RNA and associated proteins into the cytoplasm. To date, multiple host factors have been identified and proposed as attachment receptors to link virus particles to target cells; however, the details of how these interactions lead to EBOV internalization (for example, what triggers macropinocytosis) have yet to be elucidated.

Receptor tyrosine kinases (RTKs) are common cell surface receptors for many important signaling pathways that regulate cellular homeostatic process, some of which involve changes in the dynamics of actin filaments and trigger plasma membrane ruffing [16]. These events often lead to macropinocytosis, which is utilized by numerous pathogens to evade the host immune system and invade the host cells [17]. TYRO3, AXL, and MERTK (TAM) receptors are a family of tyrosine kinase receptors that have been reported to facilitate entry of various enveloped viruses, including EBOV, by functioning as attachment factors that bind to PS on the virus envelope via the serum factor Gas6 [18], which was identified by a screen of a cDNA library from cells highly susceptible to filovirus infection [9]. However, how the interaction between TAM receptors and EBOV induces macropinocytosis has not been fully investigated.

To identify new cellular targets whose inhibition attenuates EBOV infection and reveal their role in EBOV infection, we screened an RTK inhibitor library and identified RTKs that were not previously known to participate in EBOV entry.

## Results

### Inhibitor library screen to identify new RTKs that play a role in virus entry

RTKs such as the TAM receptors (AXL, TYRO3, and MERTK) are known to play a role in EBOV entry [9, 19–21]. To identify additional cellular factors that play a role in EBOV entry, we screened a library of RTK inhibitors (112 compounds), first with a recombinant, replication-competent vesicular stomatitis virus (VSV) possessing the EBOV GP in place of its own glycoprotein (VSV-EBOV GP), and then we confirmed hit compounds with our biologically contained EBOV (EBOVΔVP30) [22].

**Fig 1:**
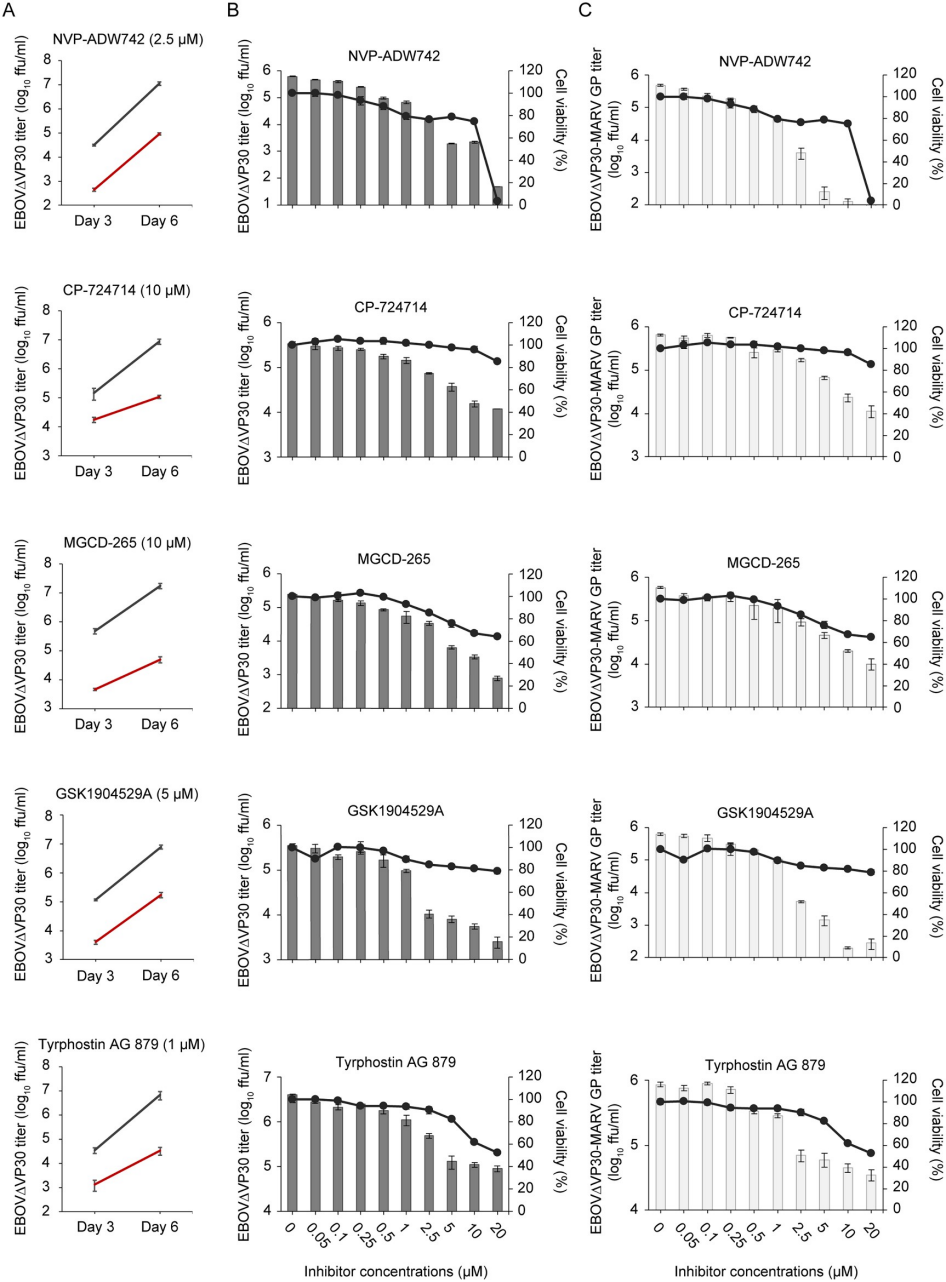
Identification of RTK inhibitors that attenuate EBOV GP- and MARV GP-mediated virus growth. (A) Titers of EBOVΔVP30-GFP from Vero VP30 cells in the presence of RTK inhibitors. Cells were treated with each RTK inhibitor at the indicated concentration (red line) or with 0.5% DMSO (black line) for 4 h prior to infection with EBOVΔVP30 at an MOI of 0.01. Virus titers were determined on days 3 and 6 post-infection. Data are presented as means ± SD of three independent experiments. (B and C) Virus titers (shown as bars) from Huh7.0 VP30 cells in the presence of RTK inhibitors. Cells were treated with the increasing doses of RTK inhibitors or with 0.5% DMSO for 4 h prior to infection with EBOVΔVP30 (B) or EBOVΔVP30-MARV GP (C) at an MOI of 0.01. Virus titers were determined on day 3 post-infection. In a separate set of experiments, viability of cells (shown as continuous lines) after treatment with inhibitors for 3 days was measured by means of a cell viability assay. Data are presented as means ± SD, and are representative of experiments performed in triplicate and repeated twice.

In the primary screen, Vero (African green monkey kidney epithelial) cells were treated for 4 h with RTK inhibitors at the highest concentration that did not reduce cell viability by more than 25% after 48 h of treatment compared to cells treated with 0.5% DMSO. After treatment, cells were infected with VSV-EBOV GP, and cell culture supernatants were collected on day 2 after infection to determine virus titers. We identified 60 inhibitors that attenuated VSV-EBOV GP growth by more than 60% (S1 Fig). We then confirmed the antiviral effect of these compounds on EBOVΔVP30 growth in Vero cells stably expressing EBOV VP30 (Vero VP30 cells) and identified the top five compounds with the best anti-EBOV activity; that is, those that caused a decrease in EBOVΔVP30 titers of more than 30-fold by day 6 after infection (Fig 1A and S2 Fig). These five compounds also inhibited EBOVΔVP30 infection of Huh7.0 VP30 cells (i.e., human hepatoma Huh7.0 cells stably expressing EBOV VP30) in a dose-dependently manner (Fig 1B). Next, we assessed whether these five inhibitors had the potential to inhibit Marburg virus (MARV) infection mediated by the MARV GP. Huh7.0 VP30 cells in the presence of each inhibitor at increasing concentrations were infected with EBOVΔVP30-MARV GP (a recombinant EBOVΔVP30 possessing the MARV GP instead of the EBOV GP). Cells treated with these inhibitors also showed a dose-dependent reduction in EBOVΔVP30-MARV GP titers (Fig 1C). The half maximal inhibitory concentration (IC_50_) and cytotoxicity concentration (CC_50_) of these inhibitors are listed in Table 1. Moreover, these inhibitors showed antiviral activity against EBOVΔVP30 viruses possessing other filovirus GPs, including the GP of recently identified filoviruses such as *Bombali ebolavirus* (Bombali virus) [23] and *Mengla dianlovirus* (Měnglà virus) [24] (S3 Fig), suggesting that these compounds have broad anti-filovirus activity (Table 2).

**Table 1:** Inhibitory concentration (IC_50_) and cytotoxicity (CC_50_) of selected compounds.

Compounds	IC_50_ (μM)		CC_50_ (μM)
	EBOVΔVP30	EBOVΔVP30-MARV GP	
NVP-ADW742	0.00211	0.0454	13.5
CP-724714	0.589	0.553	62.3
MGCD-265	0.280	0.228	26.1
GSK1904529A	0.281	0.189	105
Tyrphostin AG 879	0.220	0.434	24.7

IC_50_, 50% inhibitory concentration; CC_50_, 50% cytotoxic concentration.

**Table 2:** Effects of selected compounds on filovirus GP-mediated infection.

EBOVΔVP30 with	Inhibitory effects (log_10_) compared to DMSO-treated cells				
	NVP-ADW742	CP-724714	MGCD-265	GSK1904529A	Tyrphostin AG 879
EBOV GP	-2.5	-1.4	-1.6	-2.1	-1.5
SUDV GP	-2.7	-1.4	-1.7	-2.0	-1.5
BDBV GP	-2.6	-2.3	-1.8	-3.5	-1.3
TAFV GP	-2.8	-2.6	-2.2	-2.9	-1.4
BOMV GP	-2.4	-2.2	-2.3	-1.1	-1.5
MARV GP	-3.5	-1.7	-1.2	-3.4	-1.3
LLOV GP	-2.7	-2.2	-2.0	-3.3	-1.7
MLAV GP	-2.8	-1.7	-1.8	-1.4	-1.4
Treated concentrations	10 μM	20 μM	5 μM	20 μM	5 μM

SUDV, Sudan virus; BDBV, Bundibugyo virus; TAFV, Taï Forest virus; BOMV, Bombali virus; LLOV, Lloviu virus; MLAV, Měnglà virus.

### Effect of RTK knockdown on EBOVΔVP30 infection

**Fig 2:**
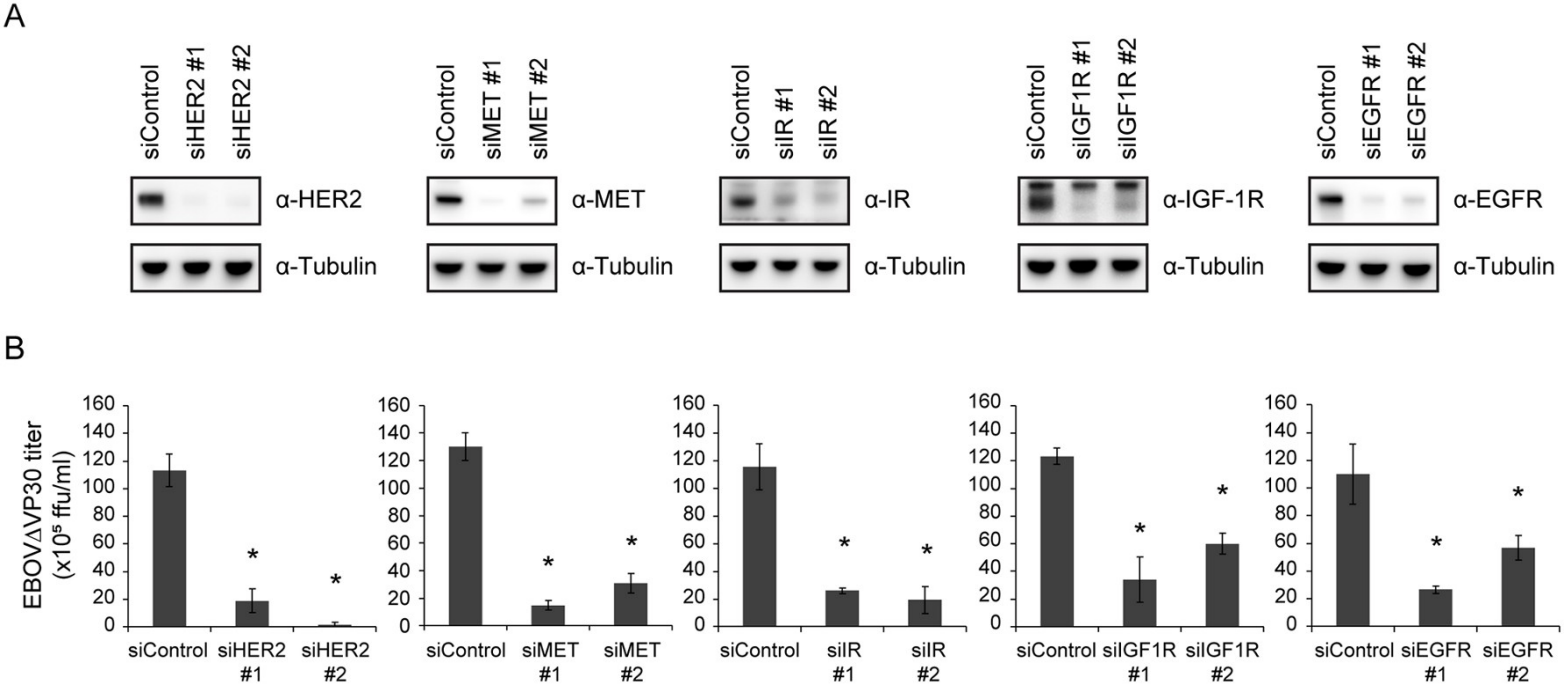
Reduction of EBOVΔVP30 growth kinetics by siRNA knockdown of RTKs. (A) RTK expression in Huh7.0 VP30 cells after siRNA knockdown. Cells were transfected with siRNAs targeting each RTK or control siRNA. On day 3 post-transfection, cells were lysed and the indicated protein expression levels were analyzed by immunoblotting. (B) Cells were transfected with siRNA as described in (A). On day 3 post-transfection, cells were infected with EBOVΔVP30 at an MOI of 0.01. Virus titers were determined on day 3 post-infection. Data are presented as means ± SD, and are representative of experiments performed in triplicate and repeated twice. (*) indicates a statistically significant difference (*p* value ≤ 0.05) from the control.

The five compounds we identified with the best anti-EBOV activity are known antagonists of the following four RTKs: human epidermal growth factor receptor 2 (HER2), mesenchymal-epithelial transition factor (MET), insulin-like growth factor 1 receptor (IGF-1R), and insulin receptor (IR) (Table 3). To further assess the involvement of these RTKs in EBOV entry, we examined virus infection after decreasing the gene expression of each RTK by transfecting Huh7.0 VP30 cells with siRNAs (two siRNAs against each RTK) that target different sites of the RTK mRNA transcript. Although no epidermal growth factor receptor (EGFR) inhibitors were hits in our screen, we included siRNAs against this RTK because EGFR-mediated signaling is reported to be used by many viruses for entry, replication, and modification of the host immune responses [25, 26]. Knockdown of gene expression of each RTK by at least one siRNA (Fig 2A) significantly attenuated EBOV**Δ**VP30 titers compared with infected cells transfected with a control siRNA (Fig 2B), suggesting that the RTKs that we identified in our screen are important for EBOV infection.

**Table 3:** Selected compounds that attenuate EBOVΔVP30.

Compounds	Target molecules
NVP-ADW742	IGF-1R
CP-724714	HER2
MGCD-265	MET
GSK1904529A	IGF-1R and IR
Tyrphostin AG 879	HER2

### EBOVΔVP30-induced phosphorylation of AKT1 is dependent on HER2

**Fig 3:**
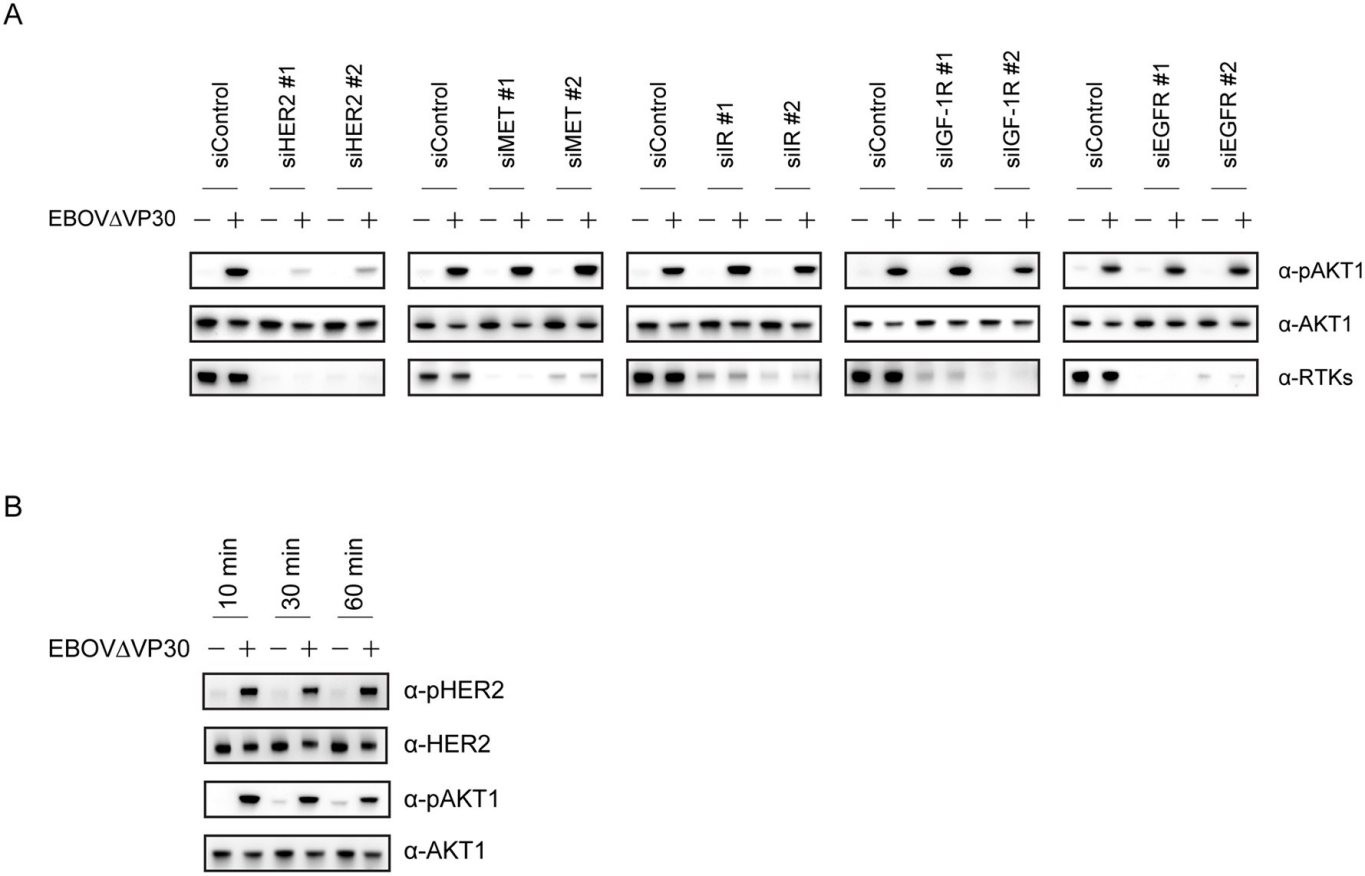
Reduction of AKT1 phosphorylation after HER2 knockdown in EBOVΔVP30 -infected cells. (A) Phosphorylation levels of AKT1 in Huh7.0 VP30 cells after RTK knockdown. Cells were transfected with siRNAs targeting each RTK or control siRNA and then on day 3 post-transfection, were infected with EBOVΔVP30 at an MOI of 3.0 for 30 min. The indicated protein expression levels were analyzed by immunoblotting. (B) Huh7.0 VP30 cells were infected with EBOVΔVP30 at an MOI of 3.0 for the noted timepoints. The indicated protein expression levels were analyzed by immunoblotting.

Saeed *et al*. demonstrated that EBOV entry requires the activation of the PI3K/AKT signaling pathway and that specific PI3K inhibitors such as LY294002 inhibit virus entry, thus reducing EBOV titers [27]. Therefore, we examined whether any of the RTKs that we identified in our screen play a role upstream of AKT1 phosphorylation during EBOVΔVP30 entry. Phosphorylation of AKT1 was detected 30 min after infection with EBOVΔVP30 in Huh7.0 VP30 cells transfected with a control siRNA (Fig 3A). AKT1 phosphorylation was also detected in infected cells transfected with siRNAs against MET, IR, IGF-1R, and EGFR (Fig 3A). Interestingly, in infected cells transfected with siRNAs against HER2, AKT1 phosphorylation was appreciably impaired (Fig 3A). Furthermore, HER2 phosphorylation was detected at 10 min post-infection and lasted for at least 60 min after infection with EBOVΔVP30 (Fig 3B). Collectively, these data suggest that HER2 mediates upstream EBOV-induced activation of the PI3K/AKT signaling pathway during entry.

### Additional evaluation of the effects of HER2 inhibitors and anti-HER2 antibodies on EBOVΔVP30 infection

To examine whether HER2 inhibitors also decrease virus infection in primary cells, we used primary human umbilical vein endothelial cells that express VP30 (HUVEC VP30). Endothelial cells were used for this evaluation because infection of this cell type by EBOV is linked to viral pathogenesis [28], and these cells express detectable levels of HER2 (S4 Fig). We found that both CP-724714 and Tyrphostin AG 879 caused a dose-dependent reduction in EBOVΔVP30 titers (S5 Fig), suggesting that the mode of inhibition is similar in cell lines and primary cells expressing HER2.

In addition to HER2 inhibitors, therapeutic monoclonal antibodies against HER2 have also been developed. The therapeutic anti-HER2 antibody Trastuzumab inhibits the binding of *Mycobacterium leprae* to cells [29] and the endocytosis of *Candida albicans* [30]. We tested whether Trastuzumab, and a second therapeutic HER2 antibody Pertuzumab, could inhibit EBOVΔVP30 infection. Huh7.0 VP30 cells were infected with EBOVΔVP30 in the presence of each antibody, individually or in combination, at 10 or 50 μg/ml. On day 3 post-infection, there was no significant decrease in EBOVΔVP30 titers between untreated and treated cells (S6 Fig). However, virus entry mediated by EBOV GP was inhibited by the antibody treatment (S7A Fig). Compared to the inhibition of virus entry induced by the HER2 inhibitors (S7B Fig), the inhibitory effect of the anti-HER2 antibodies was relatively weak, which may explain the limited inhibitory effect of these antibodies on EBOVΔVP30 growth.

### HER2 kinase activity enhances EBOV entry and macropinocytosis

**Fig 4:**
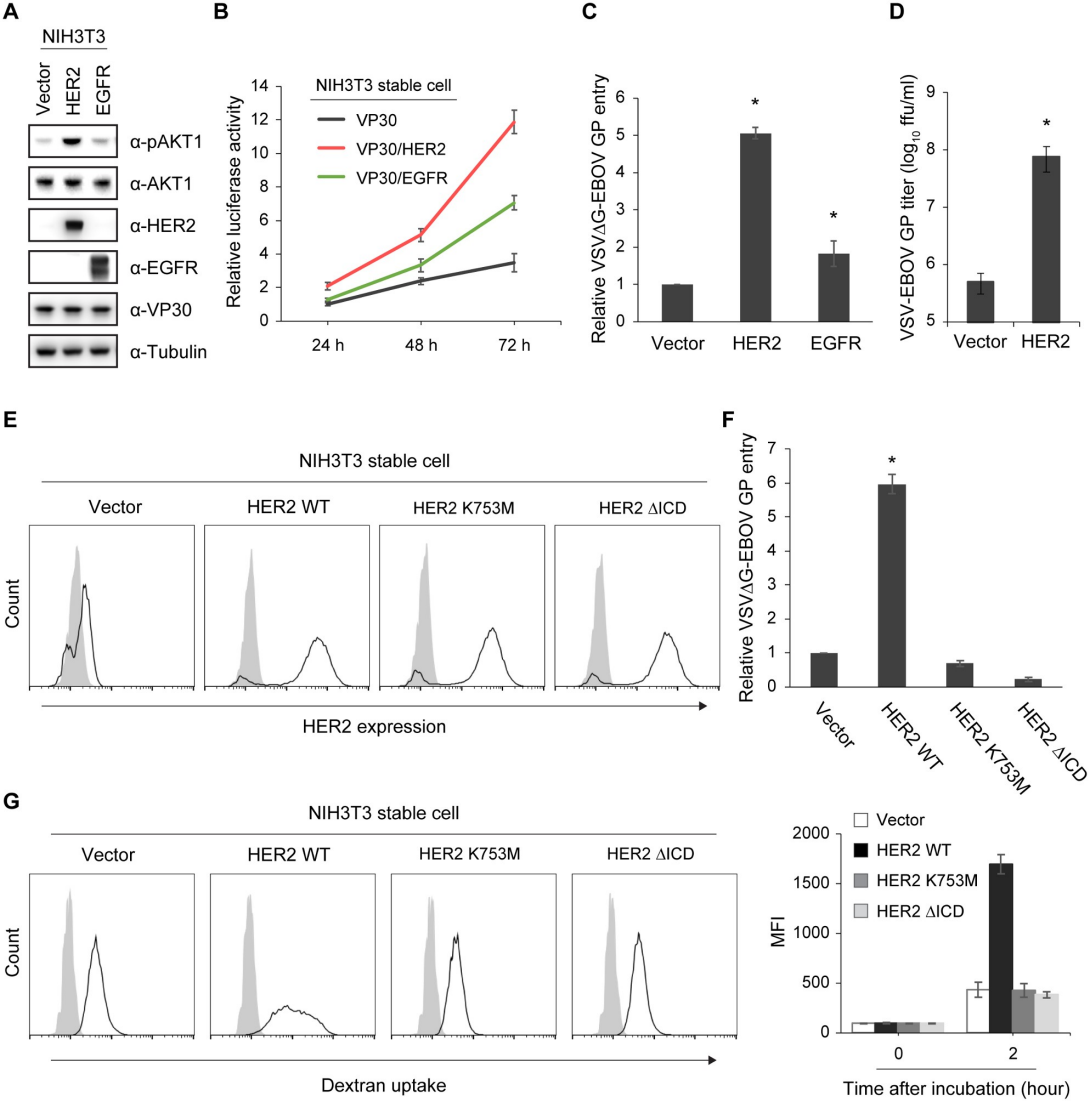
HER2 kinase-mediated enhancement of EBOV entry and macropinocytosis. (A) Phosphorylation of AKT1 in NIH3T3 VP30 cells stably expressing HER2 or EGFR. The indicated protein expression levels were analyzed by immunoblotting. (B) EBOV-driven luciferase activity in NIH3T3 VP30, NIH3T3 VP30/HER2, or NIH3T3 VP30/EGFR cell lines infected with EBOVΔVP30-luc. Virus-driven Renilla luciferase activity was measured at 24, 48, and 72 h post-infection. The relative changes compared to the luciferase activity from the NIH3T3 VP30 cell line at 24 h post-infection are presented as means ± SD. Data are representative of experiments performed in triplicate and repeated twice. (C) Relative luciferase activity in NIH3T3 stable cell lines expressing HER2 or EGFR, or in an empty vector control cell line, after infection with VSVΔG-EBOV GP virus at an MOI of 0.5. Data are presented as means ± SD, and are representative of experiments performed in triplicate and repeated at least three times. (*) indicates a statistically significant difference (*p* value ≤ 0.05) from the control. (D) Titers of VSV-EBOV GP from the NIH3T3 HER2 cell line or its empty vector control cell line infected with VSV-EBOV GP at an MOI of 0.001. Virus titers were determined on day 2 post-infection. Data are presented as means ± SD, and are representative of three independent experiments. (*) indicates a statistically significant difference (*p* value ≤ 0.05) from the control. (E) HER2 cell surface expression in NIH3T3 stable cell lines expressing either HER2 WT or the indicated kinase-deficient mutants or in an empty vector control cell line. Cells were stained with anti-HER2 antibody (black line) or a control antibody (gray shading) and analyzed by flow cytometry. (F) Relative luciferase activity in the NIH3T3 stable cell lines shown in (E) after infection with VSVΔG-EBOV GP virus at an MOI of 0.5. Data are presented as means ± SD, and are representative of experiments performed in triplicate and repeated at least three times. (*) indicates a statistically significant difference (*p* value ≤ 0.05) from the control. (G) Dextran uptake by the NIH3T3 stable cell lines shown in (E) after incubation with 400 μg/ml dextran 10K-Alexa 594 for 0 h (gray shading) or 2 h (black line). Cells were analyzed by flow cytometry. The graph on the right represents the MFI ± SD from experiments performed in triplicate and repeated twice.

Mouse NIH3T3 fibroblast cells lack detectable RTKs such as HER2 and EGFR [31, 32] and are not highly susceptible to EBOV infection [33]. Therefore, to examine the role of HER2 and EGFR in EBOV entry, we generated NIH3T3 cell lines that stably expressed VP30 (NIH3T3 VP30) or VP30 along with HER2 or EGFR (NIH3T3 VP30/HER2 and NIH3T3 VP30/EGFR, respectively). Stable expression of HER2 in the NIH3T3 VP30/HER2 cell line resulted in constitutive phosphorylation of AKT1, whereas activation of AKT1 was not observed in the NIH3T3 VP30 and NIH3T3 VP30/EGFR cell lines (Fig 4A).

Using these stable cell lines, we examined the effect of HER2 and EGFR on EBOVΔVP30 infection. The cell lines were infected with EBOVΔVP30 that expressed the Renilla luciferase reporter gene instead of the EBOV VP30 gene (EBOVΔVP30-luc) and luciferase activity was measured every 24 h after infection for 72 h. The relative luciferase activity of the infected NIH3T3 VP30/HER2 cell line at 24 h post-infection was 2-fold higher than that of the infected control NIH3T3 VP30 cell line (Fig 4B). At 72 h after infection, the relative luciferase activity of the infected NIH3T3 VP30/HER2 cells increased by 12-fold, whereas the activity of the infected control NIH3T3 VP30 cells and the NIH3T3 VP30/EGFR cells increased by only 3-fold and 6-fold, respectively (Fig 4B), suggesting that HER2 has a greater ability to enhance EBOV entry than does EGFR.

Next, we confirmed the effect of HER2 on EBOV entry by using a VSV pseudotyped with EBOV GP (VSVΔG-EBOV GP) that undergoes only a single round of replication and does not generate any progeny virus. NIH3T3 stable cell lines expressing either HER2 or EGFR or an empty vector control cell line were generated, and HER2 or EGFR protein expression was verified (S8 Fig). Entry of VSVΔG-EBOV GP was enhanced by HER2 in the NIH3T3 HER2 cell line compared to the control empty vector and the EGFR cell lines (Fig 4C). Similar results were observed with VSV-EBOV GP in the NIH3T3 HER2 cell line compared to the control cell line (Fig 4D).

The kinase domain of HER2 transduces signals downstream in various signal transduction pathways including the PI3K/AKT pathway [34]. To examine whether HER2 kinase activity is involved HER2-mediated EBOVΔVP30 entry, we generated two HER2 kinase-deficient mutant NIH3T3 cell lines. In one cell line, the lysine at position 753 in HER2 was substituted with methionine (HER2 K753M) [35]; in the other cell line, we deleted the entire HER2 intracellular kinase domain (HER2ΔICD). Cell surface expression of wild-type HER2 or the HER2 kinase-deficient mutants was similar in each cell line as determined by flow cytometry (Fig 4E). Unlike the wild-type HER2 cell lines, no constitutive phosphorylation of AKT1 was observed in either of the HER2 kinase-deficient mutant cell lines (S9 Fig). The HER2 kinase-deficient mutant cell lines did not render cells more susceptibility to VSVΔG-EBOV GP compared to the HER2 wild-type cell lines (Fig 4F), suggesting that the role of HER2 in EBOV entry depends on its kinase activity.

Our group [10] and others [11] have demonstrated that EBOV engulfment and uptake into the host cell occurs via macropinocytosis. Therefore, we next assessed whether the kinase activity of HER2 enhances macropinocytosis. To measure macropinocytotic activity, NIH3T3 HER2 cell lines (control, HER2 wild-type, and HER2 kinase-deficient mutants) were incubated with dextran 10K-Alexa 594, a fluorescent-labeled macropinocytosis marker. After a 2-h incubation, dextran uptake was measured by flow cytometry. Compared to control cells, the wild-type NIH3T3/HER2 cell line contained significantly more dextran inside the cells: a 3-fold higher mean fluorescence intensity (MFI) of dextran was observed in the wild-type NIH3T3 HER2 cell line compared with the control (Fig 4G). In the HER2 kinase-deficient mutant cell lines, there was no increase in dextran uptake compared to the control cell line (Fig 4G). These results suggest that the enhancement of EBOV entry by HER2 can be attributed to increased macropinocytotic uptake that is dependent on HER2 kinase activity.

### HER2 interacts with TAM receptors

**Fig 5:**
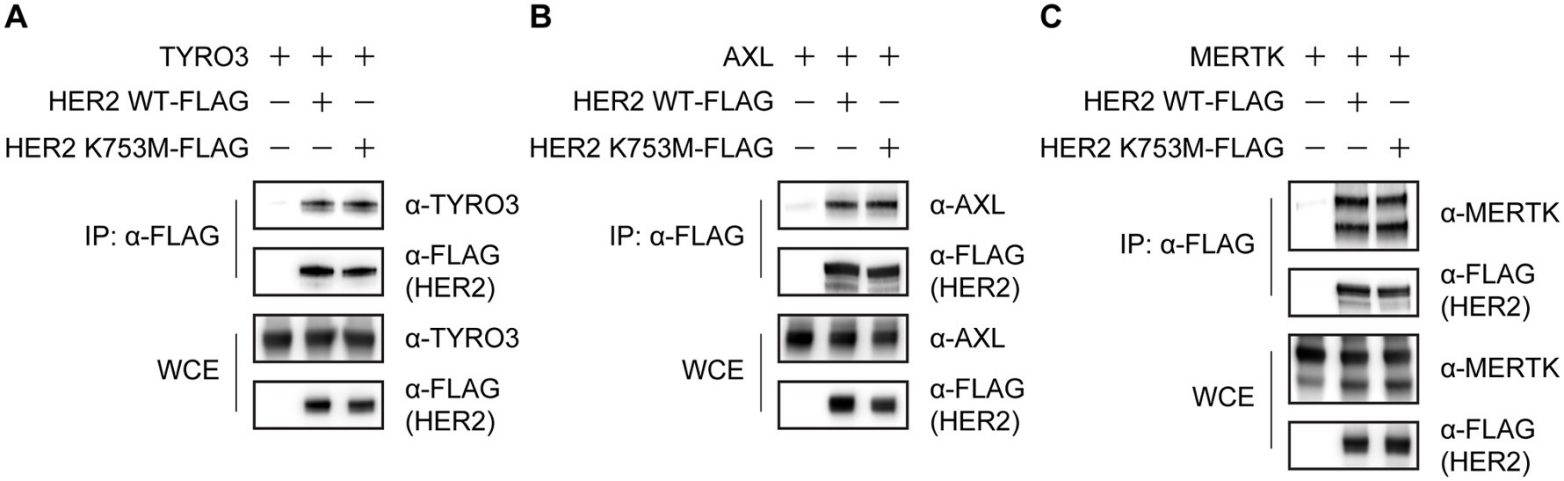
Interaction of HER2 with TAM receptors. Interaction between HER2 and TYRO3 (A), AXL (B), or MERTK (C) in HEK-293T cells transfected with the indicated combination of expression vectors. Cell lysates were immunoprecipitated with anti-FLAG antibody and then immunoblotted. Data are representative of three independent experiments. IP, immunoprecipitation. WCE, whole-cell extract.

Because HER2 has been reported to interact with the TAM receptor AXL [36], we examined whether HER2 binds to other TAM receptors (TYRO3 and MERTK) in co-immunoprecipitation assays. When each TAM receptor was overexpressed with HER2 (C-terminally FLAG-tagged; HER2 WT-FLAG), TYRO3 and MERTK, as well as AXL, were efficiently co-precipitated with HER2-FLAG (Fig 5A–5C), suggesting that HER2 interacts with all three TAM receptors and may form heterodimers with them.

The HER2 kinase-deficient mutant HER2 K753M-FLAG [35] also had similar affinity for all three TAM receptors compared to wild-type HER2 (Fig 5A–5C), suggesting that the interaction between the TAM receptors is independently of HER2 kinase activity.

### EBOVΔVP30-induced phosphorylation of HER2 is upstream of TYRO3 and MERTK

Our data that HER2 is phosphorylated during EBOVΔVP30 entry (Fig 3B) and interacts with TYRO3 and MERTK (Fig 5A and 5C), led us to examine whether endogenous TYRO3 or MERTK plays a role in the phosphorylation of HER2 during EBOVΔVP30 entry of Huh7.0 cells (we did not examine endogenous AXL because it was not detectable in Huh7.0 cells [S10 Fig]).

**Fig 6:**
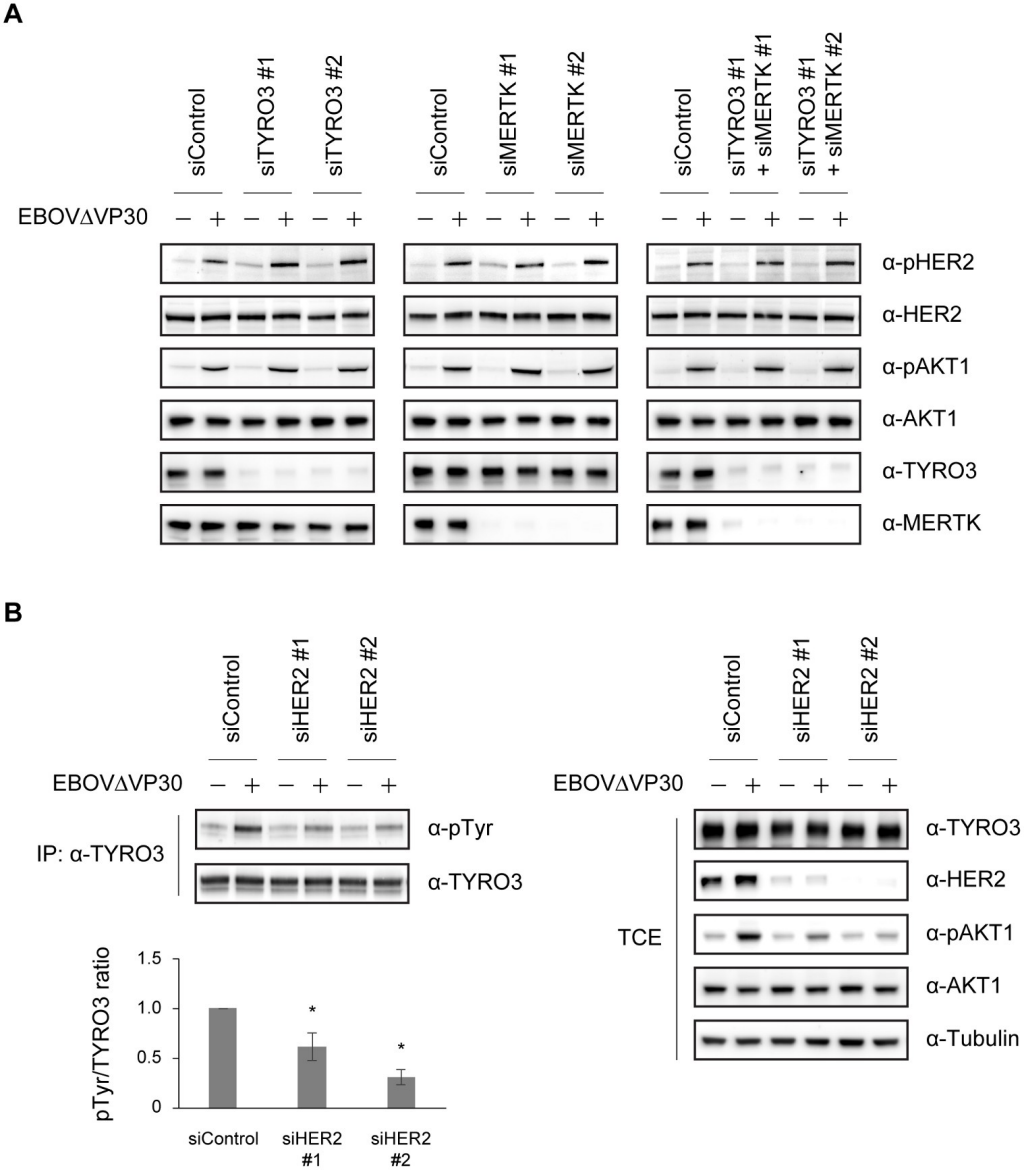
Reduced TYRO3 activation during EBOV entry after HER2 knockdown. (A) Phosphorylation level of HER2 and AKT1 in Huh7.0 cells after knockdown of either TYRO3, MERTK, or both. Cells were transfected with the indicated combination of siRNAs and then on day 3 post-transfection, were infected with EBOVΔVP30 at an MOI of 3.0 for 30 min. The indicated protein expression levels were analyzed by immunoblotting. (B) Phosphorylation level of TYRO3 in the Huh7.0 stable cell line expressing TYRO3 after HER2 knockdown. Cells were transfected with siRNAs targeting HER2 or control siRNA and then on day 3 post-transfection, were infected with EBOVΔVP30 at an MOI of 3.0 for 30 min. Cell lysates were immunoprecipitated with anti-TYRO3 antibody and then immunoblotted. Data are representative of three independent experiments. IP, immunoprecipitation. WCE, whole-cell extract. The graph represents the ratio of tyrosine-phosphorylated TYRO3 to total TYRO3 (pTyr/TYRO3) from three independent experiments. (*) indicates a statistically significant difference (*p* value ≤ 0.05) from the control.

Phosphorylation of HER2 as well as AKT1 by EBOVΔVP30 was detected at 30 min post-infection in Huh7.0 cells transfected with a control siRNA (Fig 6A; all panels). When TYRO3 or MERTK expression was reduced by siRNA treatment, there were no significant changes in HER2 and AKT1 phosphorylation levels compared to control siRNA-treated cells (Fig 6A; left and middle panels). Furthermore, even when cells were transfected with siRNAs against both TYRO3 and MERTK, no significant reduction in phosphorylation of HER2 or AKT1 was observed (Fig 6A; right panel). These data suggest that phosphorylation of HER2 is not dependent on TYRO3 or MERTK and could be done by another cell surface receptor (e.g., a phosphatidylserine receptor [18]).

Next, we investigated whether HER2 plays a role in the phosphorylation of TYRO3 during EBOVΔVP30 entry. To detect the phosphorylation of TYRO3, we generated a Huh7.0 cell line that stably expressed TYRO3 (Huh7.0 TYRO3) and performed an immunoprecipitation assay with a TYRO3 antibody for protein precipitation and a phosphotyrosine antibody for detection.

In Huh7.0 TYRO3 cells transfected with a control siRNA, the phosphorylation level of TYRO3 was increased due to EBOVΔVP30 infection compared to non-infected, control siRNA-treated cells (Fig 6B). When HER2 expression was reduced by two different siRNAs, the phosphorylation levels of TYRO3 after infection were reduced by nearly 40% and 70%, respectively, compared to the control siRNA-treated cells (Fig 6B). In addition, in the total cell extract, EBOVΔVP30-induced AKT1 phosphorylation was appreciably impaired after infection in HER2 siRNA-treated cells compared to control siRNA-treated cells (Fig 6B).

### Localization of EBOVΔVP30 particles with HER2 and TYRO3

**Fig 7:**
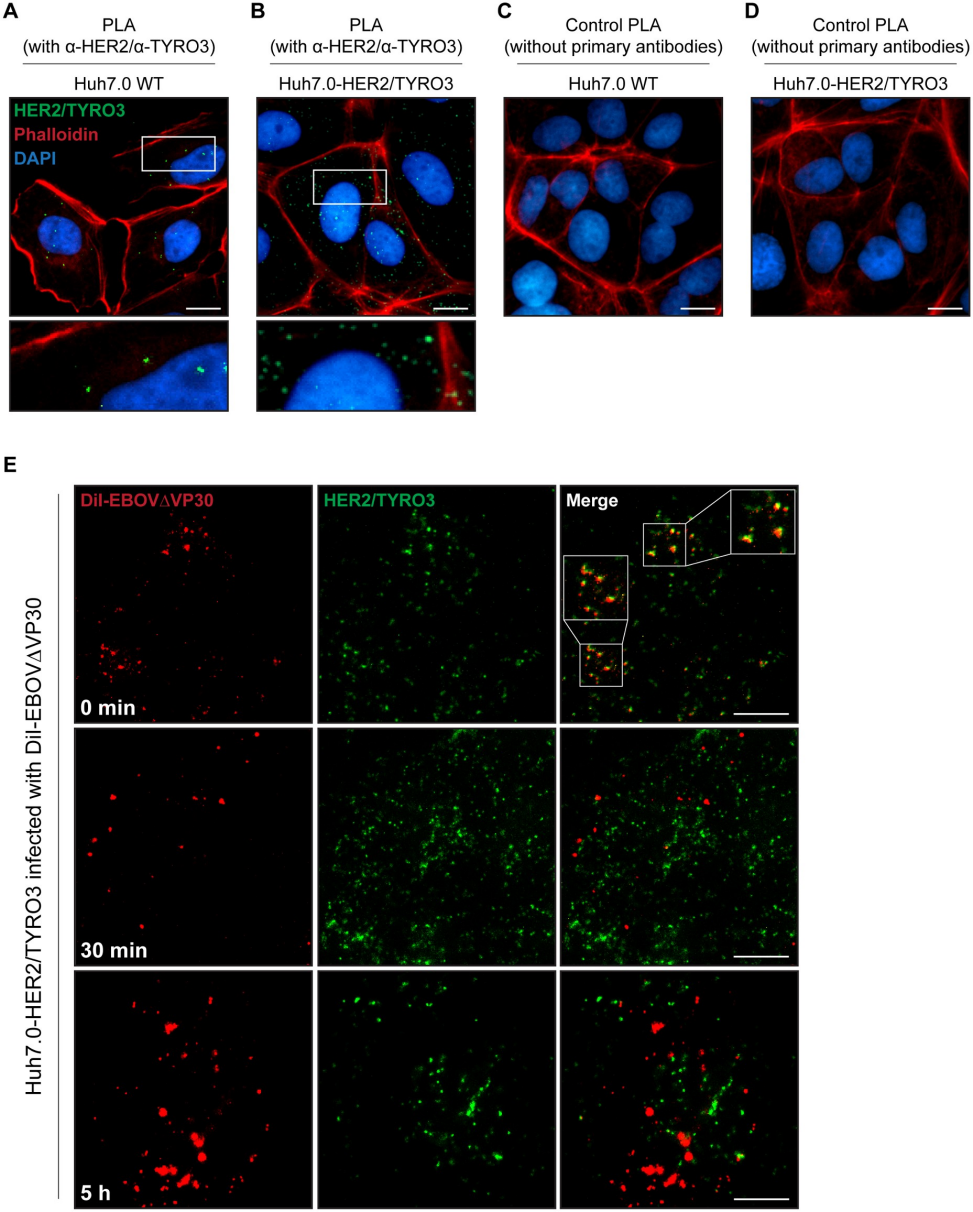
Localization of EBOVΔVP30 particles with the HER2/TYRO3 complex. Interaction between HER2 and TYRO3 in Huh7.0 WT cells (A and C) and the Huh7.0 stable cell line that overexpress both HER2 and TYRO3 (B and D). HER2/TYRO3 complexes (green) were visualized by PLA using specific antibodies (A and B). Actin and nuclei were visualized with Phalloidin (red) and DAPI (blue), respectively. The enlarged images corresponded to the boxed areas shown under the original images. Scale bars, 20 μm. (E) EBOVΔVP30 particles (red) and HER2/TYRO3 complexes (green) in the Huh7.0 HER2/TYRO3 cell line infected with DiI-labelled EBOVΔVP30 (DiI-EBOVΔVP30) for 0 min (top panels), 30 min (middle panels), or 5 h (bottom panels). HER2/TYRO3 complexes were visualized by PLA using specific antibodies. The enlarged images corresponded to the boxed areas shown under the original images. Scale bars, 20 μm.

To examine an endogenous interaction between HER2 and TYRO3 in Huh7.0 cells, we used a proximity ligation assay (PLA), a technique capable of detecting an interaction between two proteins using antibody pairs that can be visualized as individual fluorescent dots if the proteins interact. Using antibodies specific to HER2 and TYRO3, we observed co-localization of these two receptors, as indicated by green dots (~10 dots/cell) in wild-type Huh7.0 cells (Fig 7A). By comparison, in Huh7.0 cells stably expressing both HER2 and TYRO3 (Huh7.0 HER2/TYRO3), we observed more than 100 dots representing the HER2/TYRO3 complex per cell (Fig 7B, green dots). No green fluorescence was detected in either cell line when the primary antibodies against HER2 and TYRO3 were omitted from the assay (Fig 7C and 7D), demonstrating the lack of background signal in the PLA.

To track the HER2/TYRO3 complex during virus entry, we monitored EBOVΔVP30 labeled with a lipophilic membrane fluorescent dye (DiI-EBOVΔVP30) and the receptor complex over time in the Huh7.0 HER2/TYRO3 cell line. As expected, after virus was allowed to bind to the cell surface (0 min), DiI-EBOVΔVP30 particles (red) co-localized with the HER2/TYRO3 complex (green) (Fig 7E; top panels). Interestingly, this co-localization was no longer observed after the initial timepoint (0 min) where EBOVΔVP30 particles were internalized into cells (30 min) and membrane fusion occurred (5 h) (Fig 7E; middle and bottom panels) (Note: In this assay, once the DiI-labeled virus envelope fuses with the endosomal membrane, the size and intensity of the fluorescent signal are enhanced [37]). These results suggest that the HER2/TYRO3 complex may play a role in the initial internalization of the virus, but not in the delivery of the virus particles to the endosomes.

## Discussion

Here, our screen of RTK inhibitors identified five compounds that inhibited EBOVΔVP30 infection in cell culture (Table 3); whether these compounds also inhibit wild-type EBOV remains to be investigated. Two of these compounds (NVP-ADW742 and Tyrphostin AG 879) have already been reported as anti-EBOV inhibitors [38, 39]. However, here, we demonstrated that all five compounds attenuated virus entry mediated not only by the EBOV GP, but also by other filovirus GPs, indicating their potential as broad-spectrum, filovirus entry inhibitors (Tables 1 and 2).

The four cellular molecules (HER2, MET, IR, and IGF-1R) are known to be targeted by the five anti-filovirus compounds identified in our screen, but their roles in EBOV entry have not been investigated [38, 39]. While, the involvement of these four cellular targets in EBOVΔVP30 infection was confirmed in siRNA knockdown experiments (Fig 2), here, we focused on the role of HER2 in EBOV entry.

Infection of cells with EBOV results in the activation of the PI3K/AKT pathway as demonstrated by the phosphorylation of AKT1 [27]. Here, we showed that HER2 is also phosphorylated during EBOVΔVP30 infection (Fig 3B), and the downstream phosphorylation of AKT1 during infection is dependent on HER2, and not the other RTKs we evaluated (Fig 3A). In addition, macropinocytosis is enhanced by HER2 overexpression in a kinase activity-dependent manner, which results in enhancement of EBOV GP-mediated virus entry and EBOVΔVP30 growth kinetics (Fig 4).

The cell surface TAM receptors (TYRO3, AXL, and MERTK) are known to play a role in EBOV entry [9, 19–21]. It has previously been shown that HER2 interacts with AXL and is an upstream factor in the phosphorylation of AXL, although AXL has no impact on HER2 phosphorylation [36]. Our study also demonstrated an interaction between HER2 and AXL as well as an interaction between HER2 and TYRO3 and HER2 and MERTK; these interactions were independent of HER2 kinase activity since a HER2 kinase-deficient mutant (K753M) still could interact with these TAM receptors (Fig 5A–5C).

In Huh 7.0 cells, a TYRO3- and MERTK-positive cell line (S10 Fig), EBOVΔVP30 infection induced the phosphorylation of TYRO3 in a HER2-dependent manner (Fig 6), but infection did not induce phosphorylation of MERTK (S11 Fig). On examining the interaction between HER2 and TYRO3 in more detail, we observed a HER2/TYRO3 complex that included EBOVΔVP30 particles. Internalized virus particles in early endosomes are delivered to late endosomes/lysosomes in which membrane fusion occurs. Co-localization of the HER2/TYRO3 complex and the EBOVΔVP30 particles was detected only when the virus particles were on the cell surface and was no longer detected after internalization (Fig 7E), which may be because HER2 is quickly recycled to the cell surface or is resistant to internalization [40, 41]. A similar observation was reported for AXL co-localization with a pseudotyped virus with EBOV GP on its cell surface; their co-localization was not detected once the virus entered the cells [21].

The PI3K/AKT signaling pathway, which is downstream of HER2, suppresses the induction of apoptosis through both the intrinsic and extrinsic apoptotic pathways and can lead to the enhancement of cell survival in HER2-positive tumor cells [42]. For EBOV infection, both *in vitro* and animal studies, different types of cell death pathways (e.g., apoptosis, necrosis, or necroptosis) have been reported [43–45], where necrotic cell death or necroptosis rather than apoptosis occurs in EBOV-infected cells or EBOV-cultured T-cells. These observations suggest an association between the activation of HER2 and its downstream signaling pathway and the apoptosis pathways.

TAM receptor activation induces Suppressor Of Cytokine Signaling 1 (SOCS1) and SOCS3 expression through an interaction with interferon (IFN) receptors, serving as a negative feedback mechanism of IFN signaling [46]. During EBOV entry, SOCS1 and SOCS3 are upregulated through the binding of EBOV GP and Toll-like receptor 4 (TLR4) [47, 48]. It will be interesting to further investigate whether HER2 mediates the induction of SOCS1 and SOCS3 via TAM receptors or TLR4 directly or indirectly, which might lead to suppression of an anti-viral state.

To date, there have been no reports HER2 functioning as a cellular factor for virus entry. But other pathogens do exploit HER2 and its downstream signaling pathway for their entry into host cells. For example, the fungus *Candida albicans* interacts with HER2 by its invasins and stimulates phosphorylation of HER2, resulting in the endocytosis of this organism [30]. *Neisseria gonorrhoeae* and *N*. *meningitidis* also stimulate HER2 phosphorylation resulting in the accumulation of HER2 at the site of bacterial adherence, which enhances intracellular signaling leading to bacterial internalization [49, 50]. *Mycobacterium leprae* activates HER2-mediated ERK1 and ERK2 signaling by direct binding to HER2, which cause demyelination in neurodegenerative diseases [29]. With EBOV, direct binding of the virus to HER2 most likely does not occur; therefore, the downstream signaling pathways activated during EBOV entry may be different from those involved in the entry of these other organisms.

While high expression of HER2 is reported in many breast, ovarian, and gastric cancer cells [51], medium or low HER2 expression is predicted in a broad range of tissues (including EBOV antigen-positive tissues in infected animals), such as liver, lung, kidney, gastrointestinal tract, heart, skin, and immune-privileged sites such as the brain, eye, and reproductive tract [28, 52, 53]. This may suggest that HER2 has a role in the broad tissue tropism of EBOV during infection.

**Fig 8:**
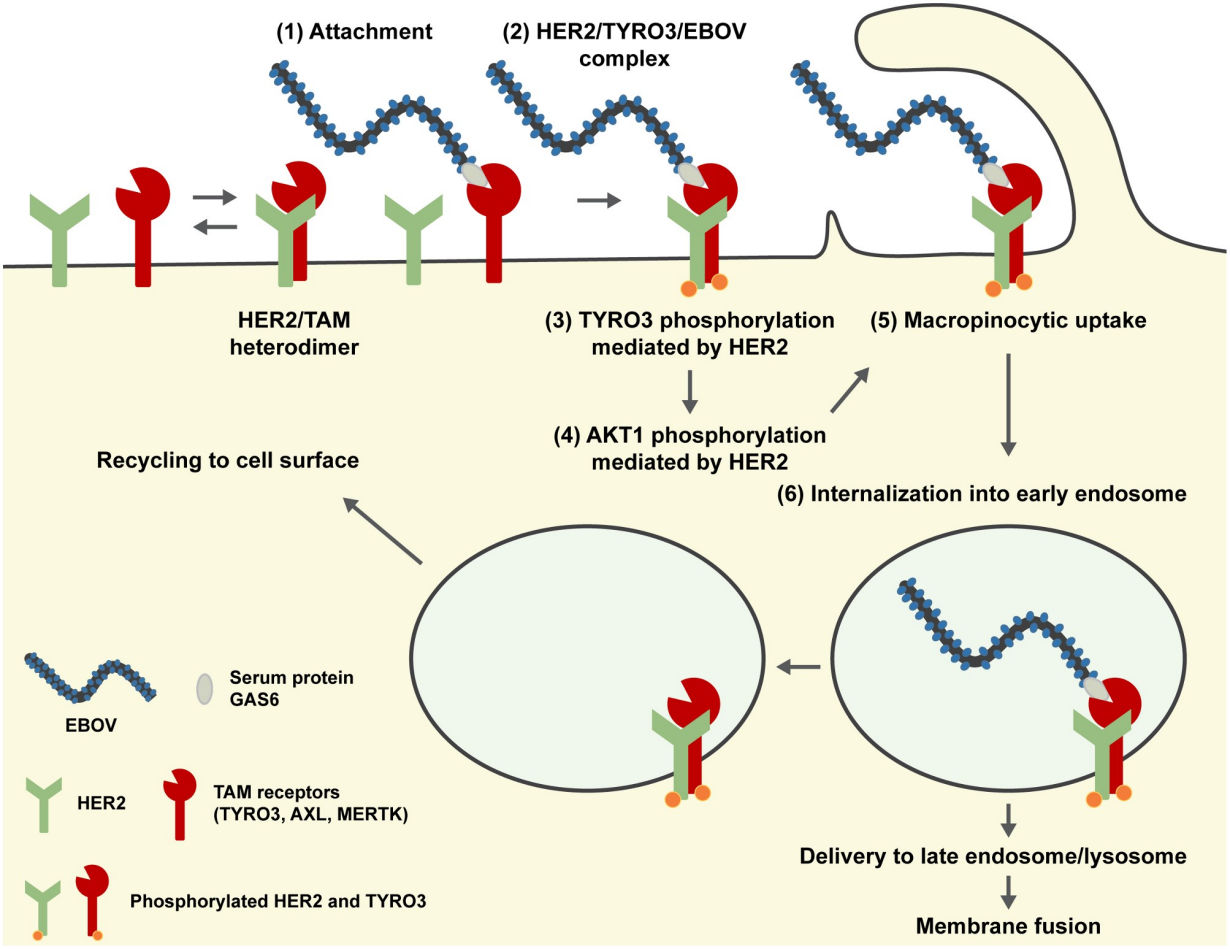
Proposed model of EBOV entry into cells expressing HER2 and TYRO3. HER2 interacts with all members of the TAM (TYRO3, AXL, and MERTK) receptor family to form heterodimers. In TAM receptor-mediated cellular entry, EBOV particles attach to the cell surface by binding to a TAM receptor (e.g., TYRO3) via the serum protein GAS6 (1). HER2 also interacts with the virus-bound TYRO3, forming a HER2/TYRO3/EBOV complex on the cell surface (2). The formation of this complex leads to HER2 phosphorylation (either autophosphorylation or phosphorylation by an as yet unknown cellular factor) and the phosphorylation of TYRO3 by HER2 (3), leading to downstream activation of PI3K/AKT signaling pathway (HER2-mediated AKT1 activation) (4) and subsequent induction of macropinocytosis (5). After internalization of the HER2/TYRO3/EBOV complexes into early endosomes via macropinocytosis (6), the complexes are disassociated, and only EBOV particles are delivered to late the endosomes/lysosomes, where later events such as membrane fusion occur. HER2 and TYRO3 are most likely recycled back to the cell surface.

In summary, although our findings should be confirmed with wild-type EBOV, we identified HER2 as a new cellular factor involved in the entry of EBOV that interacts with known cell surface receptors (TAM receptors; TYRO3, AXL, and MERTK) and demonstrated that HER2 activation induced by EBOV infection leads to downstream activation of AKT1 in the PI3K/AKT signaling pathway followed by virus uptake into the cell via macropinocytosis (Fig 8).

## Materials and methods

### Cells

Vero cells (African green monkey kidney cell line) and Vero VP30 cells (Vero cells stably expressing EBOV VP30 [22]) were grown in Eagle’s minimal essential medium (MEM) supplemented with 10% fetal bovine serum (FBS), L-glutamine, vitamins, nonessential amino acid solution, and antibiotics.

Huh7.0 VP30 cells (human hepatocarcinoma Huh7.0 cell line stably expressing EBOV VP30) were established in a similar manner as Vero VP30 cells [22]. Huh7.0 TYRO3 cells, Huh7.0 MERTK cells, and Huh7.0 HER2/TYRO3 cells (Huh7.0 cell lines stably expressing either TYRO3 or MERTK or both HER2 and TYRO3) were generated as follows: a cDNA fragment encoding each gene was cloned into the murine leukemia virus (MLV)-based retroviral vector pMXs-IRES-Neo (pMXs-IN) or pMXs-IRES-Puro (pMXs-IP) (Cell Biolabs). To generate the retrovirus, Plat-GP cells (Cell Biolabs) were co-transfected with pMXs-IN vectors encoding TYRO3 or MERTK or with the pMXs-IP vector encoding HER2 (GenBank accession no. NM_004448.3) along with an expression vector for VSV G by using Lipofectamine 2000 (Invitrogen). Two days later, the culture supernatants containing the retroviruses were collected, clarified through 0.45-μm-pore filters, and then used to transduce Huh7.0 cells. Stable cells were selected with 500 μg/ml G418 (InvivoGen) with or without 2 μg/ml puromycin (InvivoGen). The open reading frame of TYRO3 (pDONR223-TYRO3; Cat# 23916) and MERTK (pDONR223-MERTK; Cat# 23900) genes were obtained from Addgene.

NIH3T3 HER2 cells, NIH3T3 EFGR cells, NIH3T3 VP30/HER2 cells, and NIH3T3 VP30/EFGR cells (mouse fibroblast NIH3T3 cell lines stably expressing either HER2 or EGFR together with or without EBOV VP30) were generated in a similar manner as above: the pMXs-IN vector encoding EBOV VP30 and the pMXs-IP vectors encoding HER2 or EGFR (GenBank accession no. NM_005228.5) were used to generate the retrovirus. Stable cells were selected with 500 μg/ml G418 (InvivoGen) with or without 2 μg/ml puromycin (InvivoGen).

HUVEC VP30 cells (human umbilical vein endothelial cells stably expressing EBOV VP30) were generated in a similar manner as NIH3T3 VP30 cells using the pMXs-IN vector encoding EBOV VP30. Stable cells were selected with 500 μg/ml G418 (InvivoGen).

All Huh7.0 cell lines (wild-type or stable cells), NIH3T3 cell lines (wild-type or stable cells), and HEK-293T cells (human embryonic kidney cell line) were grown in high-glucose Dulbecco’s modified Eagle’s medium (DMEM) containing 10% FBS, L-glutamine, and antibiotics. HUVEC VP30 cells were grown in Endothelial Cell Growth Medium (Cell Applications, Inc.). All cells were maintained at 37°C and 5% CO_2_.

### Viruses

EBOVΔVP30 viruses expressing neomycin (EBOVΔVP30), GFP (EBOVΔVP30-GFP) or Renilla luciferase (EBOVΔVP30-luc) instead of the viral VP30 gene, along with different filovirus glycoproteins, were generated as previously described [22]. Viruses were propagated in Vero VP30 cells by using propagation medium similar to cell growth medium, but supplemented with only 2% FBS. The use of EBOVΔVP30 viruses in BSL-2 containment at the University of Wisconsin is approved by the Institutional Biosafety Committee, the NIH, and the CDC. A replication-incompetent VSV possessing the firefly luciferase gene in place the VSV G gene and pseudotyped with EBOV GP (VSVΔG-EBOV GP) and replication-competent VSV possessing the EBOV GP (VSV-EBOV GP) were prepared as previously described, respectively [54, 55].

### Cell viability assay

Cells were seeded in triplicate into 96-well plates (2.0 x 10^4^ cells/well) and then treated with each RTK inhibitor over a concentration range of 0.125–20 μM or with 0.5% DMSO as a control for 24–48 h, or were treated with anti-HER2 antibodies at 10 or 50 μg/ml for 3 days. Cell viability was assessed by using Cell Titer-Glo (Promega) according to the manufacturer’s protocol.

### RTK inhibitor screen

#### Cytotoxicity test

An RTK inhibitor library of 112 compounds was obtained from Selleckchem. To test cytotoxicity, Vero cells treated for 24 h and 48 h with inhibitors at 5 μM were assessed by use of a cell viability assay. The cytotoxicity test was repeated at least twice. Inhibitors that resulted in less than 75% cell viability compared to the control cells were tested at lower concentrations (e.g., 2.5, 1.0, 0.5, 0.25, or 0.125 μM) until their presence resulted in more than 75% cell viability. Inhibitors that led to more than 75% cell viability compared to the control cells were tested at higher concentrations (10 and 20 μM).

#### The primary screen

Vero cells were seeded in duplicate into 24-well plates (1.0 x 10^5^ cells/well) and then treated with the inhibitors at the highest concentration that did not reduce cell viability by more than 25% over a 48-h treatment compared to the control cells. After 4 h of treatment, cells were infected with VSV-EBOV GP at an MOI of 0.001 and then cultured in the presence of inhibitors for 2 days. Viral titers were determined by means of a focus-forming assay as previously described [22]. Inhibitors that attenuated VSV-EBOV GP growth by more than 60% were selected for the second screen.

#### The secondary screen

Vero VP30 cells were seeded in duplicate into 24-well plates (1.0 x 10^5^ cells/well) and then treated with the selected inhibitors at the same concentrations used in the primary screen or at 5–20 μM when the inhibitors did not show cytotoxicity at any of these concentrations. After 4 h of treatment, cells were infected with EbolaΔVP30 at an MOI of 0.001 and then cultured in the presence of the inhibitors. Supernatants were harvested on days 3 and 6 post-infection, and viral titers were determined by means of a focus forming assay as previously described [22].

### Assessment of viral titers

To validate the antiviral activity of the selected five compounds on the infection of Huh7.0 VP30 cells and the antiviral activity of HER2 inhibitors on the infection of HUVEC VP30 cells, cells were seeded in triplicate into 24-well plates (1.0 x 10^5^ cells/well) and then treated with inhibitors at 0.05–20 μM 4 h prior to infection with EBOVΔVP30-GFP or EBOVΔVP30 bearing other filovirus GPs at an MOI of 0.01–0.002. After being washed twice, the cells were cultured in the presence of inhibitors for 3 days. Viral titers were determined by means of a focus forming assay as previously described [22].

For the evaluation of the anti-HER2 antibodies, Huh7.0 VP30 cells were seeded in triplicate into 24-well plates (1.0 x 10^5^ cells/well) and then treated with Trastuzumab (Selleckchem), Pertuzumab (Selleckchem), or both at 10 or 50 μg/ml 1 h prior to infection with EBOVΔVP30-GFP at an MOI of 0.01. After being washed twice, the cells were cultured in the presence of the antibodies for 3 days. Viral titers were determined by performing a focus forming assay.

To determine the susceptibility of the NIH3T3 stable cells to EBOVΔVP30 and VSV-EBOV GP, the cells were seeded in triplicate into 96-well plates (3.0 x 10^4^ cells/well) for EBOVΔVP30-luc infection and seeded in duplicate into 24-well plates (1.5 x 10^5^ cells/well) for VSV-EBOV GP infection. Cells were then infected with EBOVΔVP30-luc or VSV-EBOV GP at an MOI of 0.001. Virus-driven Renilla luciferase activity was measured on days 1–3 post-infection by using EnduRe Live Cell Substrate (Promega) according to the manufacturer’s instructions. Supernatants containing VSV-EBOV GP were collected on day 2 post-infection and virus titers were determined.

### Transfection of siRNA

Human siRNAs for HER2 (4390824_s613 and 4392420_s612, Ambion), MET (6618 and 6622, Cell signaling technology), IR (AM51331_29 and AM51331_31, Ambion), IGF-1R (AM51331_110754 and 4392420_s223917, Ambion), EGFR (6480 and 6482, Cell signaling technology), TYRO3 (Hs_TYRO3_5 and Hs_TYRO3_6, Qiagen), MERTK (Hs_MERTK_5 and Hs_MERTK_11, Qiagen), and a negative control siRNA (All Star Negative Control siRNA, Qiagen) were purchased. Cells were seeded in triplicate into 24-well plates (1.0 x 10^5^ cells/well) or seeded into 60-mm dishes (1.0 x 106 cells/dish) 1–2 h prior to transfection. Each siRNA was transfected into cells at a final concentration of 10 nM by using the Lipofectamine RNAiMAX reagent (Invitrogene). On day 3 post-infection, cells were evaluated for knockdown efficiency by western blot analysis or EBOVΔVP30 infection.

### Western blot analysis

Cells were washed with PBS and treated with lysis buffer (1% NP-40, 50 mM Tris-HCl [pH 7.4], 150 mM NaCl, 0.25% sodium deoxycholate, with/without 0.1% SDS) containing protease inhibitor cocktail (Sigma). Cell lysates were mixed with an equal volume of 2x Laemmli sample buffer (Bio-Rad) containing 5% β-mercaptoethanol and then incubated at 95°C for 10 min. Samples were resolved by SDS-PAGE onto a 4%–20% SDS-polyacrylamide gel (Life Technologies). After electrophoresis, proteins were transferred onto a polyvinylidenedifluoride membrane (Life Technologies) and blocked for 1 h at room temperature with 5% skim milk/TBS solution containing 0.05% Tween-20 (TBS-T; Sigma). Membranes were incubated overnight at 4°C with primary antibodies against HER2 (2165, Cell signaling technology), phospho-HER2 (Tyr1248) (2247, Cell signaling technology), MET (3148, Cell signaling technology), IGF-1R (3018, Cell signaling technology), IR (3020, Cell signaling technology), EGFR (4267, Cell signaling technology), AKT1 (2938, Cell signaling technology), phosphor-AKT1 (Ser473) (9018, Cell signaling technology), TYRO3 (5585, Cell signaling technology), AXL (8661, Cell signaling technology), MERTK (4319, Cell signaling technology), phospho-tyrosine (9411, Cell signaling technology), alpha-tubulin (ab7291, Abcam), FLAG tag (M2, Sigma), and EBOV VP30 (cl. 3). Membranes were then incubated with a secondary antibody coupled to horseradish peroxidase for 1 h. Bound antibody was detected with SuperSignal Pico, Dura, or Femto chemiluminescence reagent (Thermo Fisher Scientific) and a FluorChem HD2 imager (Alpha Innotech).

### Assessment of cell entry

To examine EBOV GP-mediated cellular entry, NIH3T3 stable cells were seeded in triplicate into 96-well plates (3.0 x 10^4^ cells/well) and then infected with VSVΔG-EBOV GP, which was treated with a neutralizing monoclonal antibody against the VSV G protein (cl. I-1) to abolish the background infectivity of the parental VSV-G virus in the virus stock of VSVΔG-EBOV GP. Twenty-four hours later, cells were lysed and firefly luciferase activity was measured by using the Steady-Glo luciferase assay system (Promega) according to the manufacturer’s instructions.

To evaluate the antiviral effect of HER2 inhibitors and anti-HER2 antibodies on EBOV GP-mediated cellular entry, Huh7.0 VP30 cells were seeded in triplicate into 96-well plates (2.0 x 10^4^ cells/well) and then treated with the inhibitors for 4 h or with the antibodies for 1 h prior to infection with VSVΔG-EBOV GP. Luciferase activity in infected cells was measured as described above.

### Flow cytometric analysis

NIH3T3 stable cells were detached with 0.25% trypsin, washed with PBS containing 2% FBS (PBS-2% FBS), and fixed with 4% paraformaldehyde for 15 min at room temperature. Cells were then incubated with mouse anti-HER2 antibody (MAB1129, R&D Systems) or mouse isotype control antibody (5415, Cell signaling technology) for 1 h at room temperature. After being washed with PBS-2% FBS twice, the cells were incubated with anti-mouse IgG–Alexa Fluor 488 (A11029, Molecular Probes) for 30 min at room temperature. After several washes, the cells were analyzed with a FACS Aria III flow cytometer (BD Biosciences) and FlowJo software (Tree Star).

### Dextran uptake assay

NIH3T3 stable cells grown in triplicate in 24-well plates (1.5 x 10^5^ cells/well) were washed with phenol red-free DMEM (Gibco Life Technologies) containing 2% FBS and 4% BSA and then incubated in the same medium containing 400 μg/ml dextran10K-Alexa Fluor 594 (Invitrogen) for 0 h or 2 h at 37 °C. After they were washed with PBS, the cells were detached with trypsin and fixed with 4% paraformaldehyde for 15 min at room temperature. After several washes, the fluorescence intensities in the cells were analyzed by flow cytometry.

### Co-immunoprecipitation assay

The HER2 WT and HER2 K753M genes were C-terminally tagged with FLAG and inserted into a pCAGGS expression vector. The open reading frames of the TYRO3 (pDONR223-TYRO3; 23916), AXL (pDONR223-AXL; 23945), and MERTK (pDONR223-MERTK; 23900) genes were obtained from Addgene and cloned into a pCAGGS expression vector.

For co-IP in HEK-293T cells transfected with the indicated expression vectors using TransIT-LT1 (Mirus) for two days, the cells were washed once with PBS and treated for 30 min with lysis buffer (0.1% SDS, 1% NP-40, 50 mM Tris-HCl [pH 7.4], 150 mM NaCl, 0.25% sodium deoxycholate) containing protease inhibitor cocktail (Sigma) to prepare whole-cell extract (WCE). Cell extracts were mixed with pre-conjugated anti-FLAG M2 magnetic beads (Sigma) and incubated on a rotator at 4 °C overnight. The next day, the magnetic beads were washed three times with lysis buffer. The bound proteins were eluted with excess FLAG peptide (Sigma) and analyzed by SDS-PAGE followed by immunoblotting with the indicated antibodies.

For co-IP in Huh7.0 stable cells under siRNA treatment followed by EBOVΔVP30 infection at an MOI of 3.0 for 30 min, the cells were washed once with PBS and treated for 30 min with lysis buffer (0.1% SDS, 1% NP-40, 50 mM Tris-HCl [pH 7.4], 150 mM NaCl, 0.25% sodium deoxycholate) containing protease inhibitor cocktail (Sigma) to prepare WCE. Cell extracts were mixed with anti-TYRO3 antibody (5585, Cell signaling technology) or anti-MERTK antibody (4319, Cell signaling technology) with protein A/G magnetic beads (Pierce) and incubated on a rotator at 4 °C overnight. The next day, the magnetic beads were washed three times with lysis buffer. The bound proteins were incubated in the sample buffer at 95°C for 10 min and analyzed by SDS-PAGE followed by immunoblotting with the indicated antibodies.

### Preparation of fluorescence-labeled EBOVΔVP30 particles

The culture supernatant containing EBOVΔVP30-neo from Vero VP30 was harvested and centrifuged at 3,500 rpm for 15 min to remove cell debris. Viral particles were then precipitated through 20% sucrose by centrifugation at 13,000 rpm for 1.0 h at 4 °C using an SW32 rotor (Beckman Coulter). The pellet was then resuspended in PBS and fractionated through a 20%–50% sucrose gradient in PBS at 28,000 rpm for 2.5 h at 4°C using an SW41 rotor (Beckman Coulter). The fractionated viral particles were fluorescently labeled with the lipophilic tracer CM-DiI (Molecular Probes) by incubating them in 5 μM CM-DiI solution in the dark for 1 h at room temperature, followed by centrifugation at 27,000 rpm for 2.0 h at 4°C to remove excess dye. The pellet was resuspended in PBS and stored at -80°C until use.

### Imaging of DiI-labeled EBOVΔVP30 during cell entry

Huh7.0 stable cells grown on 8-well Lab-tek chamber slides (Thermo Fisher Scientific) were washed with phenol red-free DMEM (Gibco Life Technologies) containing 2% FBS and 4% BSA and incubated with DiI-labeled EBOVΔVP30 (3–5 μg/well) in the same medium for 30 min at room temperature. The cells were then washed with the same medium to remove the unbound virions and incubated for 0 min, 30 min, or 5 h at 37°C. After fixation with 4% paraformaldehyde for 15 min at room temperature, the samples were subjected to a proximity ligation assay and then analyzed using an LSM 510 META confocal microscope (Carl Zeiss) with ZEN 2009 software (Carl Zeiss).

### Detection of HER2/TYRO3 complex by proximity ligation assay (PLA)

Huh7.0 cells (wild-type or stable cells) grown on 8-well Lab-tek chamber slides (Thermo Fisher Scientific) were fixed with 4% paraformaldehyde for 15 min at room temperature, permeabilized with 0.005% Triton X-100 for 10 min, and then subjected to the in situ PLA using the Duolink Detection kit (Sigma) according to the manufacturer’s protocol with slight modifications. Briefly, after blocking, cells were incubated overnight at 4°C with the following primary antibodies: mouse anti-HER2 antibody (1:100; MAB1129, R&D Systems) and rabbit anti-TYRO3 antibody (1:100; 5585, Cell signaling technology). Incubation with PLA plus and minus probes (a pair of oligonucleotide-labeled secondary antibodies) and ligation (formation of a closed, circle DNA template) were performed as described in the manufacturer’s protocol. Amplification of the circled DNA template (rolling circle amplification) was performed for 180 min. Then, nuclei and F-actins were stained with 3 μM 4’,6-diamidino-2-phenylindole (DAPI) and 165 nM phalloidin-Alexa Fluor 546 (Invitrogen), respectively, for 60 min. Protein complexes were visualized by using an LSM 510 META confocal microscope (Carl Zeiss) with ZEN 2009 software (Carl Zeiss) or an EVOS FL microscope (Thermo Fisher Scientific). Negative controls were performed by excluding the primary antibodies.

## Statistical analysis

Statistical analysis was conducted using R software (version 3.3.1). For all data, the Student’s two-tailed, paired, and unpaired t-test was used to assess statistical differences between samples. Significance levels were set at *p* ≤ 0.05.

## Supporting information

S1 Fig Primary screen for RTK inhibitors that attenuate VSV-EBOV GP infection. Dot plot of the inhibition of VSV-EBOV GP titers after treatment with RTK inhibitors. Vero cells were treated with each RTK inhibitor at the indicated concentration for 4 h prior to infection with VSV-EBOV GP at an MOI of 0.001. Virus titers were determined on day 2 post-infection and compared to those in control cells treated with 0.5% DMSO. Data are presented as means of at least two independent experiments.
(TIF)

S2 Fig Secondary screen for RTK inhibitors that attenuate EBOVΔVP30 growth kinetics. Heat map of fold changes in EBOVΔVP30 titers in the presence of RTK inhibitors. Vero VP30 cells were treated with each RTK inhibitor at the indicated concentration for 4 h prior to infection with EBOVΔVP30 at an MOI of 0.001. Virus titers were determined on days 3 and 6 post-infection and compared with those in the control cells treated with 0.5% DMSO. Data are presented as fold changes of means from at least three independent experiments.
(TIF)

S3 Fig Effects of selected RTK inhibitors on EBOVΔVP30 infection mediated by other filovirus GPs. Titers of chimeric EBOVΔVP30 bearing the indicated filovirus GPs from infected Huh7.0 VP30 cells in the presence of RTK inhibitors. Cells were treated with each RTK inhibitor at the indicated concentration or with 0.5% DMSO for 4 h prior to infection with the viruses at an MOI of 0.01–0.002. Virus titers were determined on day 3 post-infection. Data are presented as means ± SD, and are representative of experiments performed in triplicate and repeated twice. SUDV, Sudan virus; BDBV, Bundibugyo virus; TAFV, Taï Forest virus; BOMV, Bombali virus; LLOV, Lloviu virus; MLAV, Měnglà virus.
(TIF)

S4 Fig HER2 expression in primary human endothelial cells. HER2 expression in HUVEC VP30 and Huh7.0 VP30 cells. The indicated protein expression levels were analyzed by immunoblotting.
(TIF)

S5 Fig Effect of HER2 inhibitors on EBOVΔVP30 infection in primary cells. Titers of EBOVΔVP30-GFP (shown as bars) from HUVEC VP30 cells in the presence of the HER2 inhibitors CP-724714 (A) and Tyrphostin AG 879 (B). Cells were treated with increasing doses of the indicated inhibitors or with 0.5% DMSO for 4 h prior to infection with EBOVΔVP30 at an MOI of 0.005. Virus titers were determined on day 3 post-infection. In a separate set of experiments, cell viability (shown as continuous lines) after treatment with inhibitors for 3 days was measured by performing a cell viability assay. Data are presented as means ± SD, and are representative of experiments performed in triplicate and repeated twice.
(TIF)

S6 Fig Effect of therapeutic anti-HER2 antibodies on EBOVΔVP30 infection. Titers of EBOVΔVP30-GFP (shown as bars) from Huh7.0 VP30 cells in the presence of the anti-HER2 antibodies Trastuzumab (A), Pertuzumab (B), and a combination of both (C). Cells were treated with the indicated concentrations of the antibodies for 1 h prior to infection with EBOVΔVP30 at an MOI of 0.01. Virus titers were determined on day 3 post-infection. In a separate set of experiments, cell viability (shown as continuous lines) after treatment with antibodies for 3 days was measured by performing a cell viability assay. Data are presented as means ± SD, and are representative of experiments performed in triplicate and repeated twice.
(TIF)

S7 Fig Effect of therapeutic anti-HER2 antibodies and HER2 inhibitors on EBOV GP-mediated virus entry. (A) Relative luciferase activity in Huh7.0 VP30 cells in the presence of the indicated anti-HER2 antibodies after infection with VSVΔG-EBOV GP virus at an MOI of 0.5. Data are presented as means ± SD of four independent experiments performed in triplicate. (*) indicates a statistically significant difference (*p* value ≤ 0.05) from the control. (B) Relative luciferase activity in Huh7.0 VP30 cells in the presence of the indicated HER2 inhibitors after infection with VSVΔG-EBOV GP virus at an MOI of 0.5. Data are presented as means ± SD of three independent experiments performed in triplicate. (*) indicates a statistically significant difference (*p* value ≤ 0.05) from the control.
(TIF)

S8 Fig HER2 and EGFR expression in stable cell lines. HER2 and EGFR expression in NIH3T3 stable cell lines expressing either HER2 or EGFR. The indicated protein expression levels were analyzed by immunoblotting.
(TIF)

S9 Fig AKT1 activation in stable cell lines. Phosphorylated AKT1 in NIH3T3 stable cell lines expressing either HER2 WT or the indicated kinase-deficient mutants or in an empty vector control cell line. The indicated protein expression levels were analyzed by immunoblotting. The numbers indicate two different stable cell line populations generated in the same setting.
(TIF)

S10 Fig Expression of TAM receptors in cell lines. Expression of TYRO3, AXL, and MERTK in Vero and Huh7.0 cells. The indicated protein expression levels were analyzed by immunoblotting.
(TIF)

S11 Fig Phosphorylation level of MERTK during EBOV entry. The phosphorylation level of MERTK in Huh7.0 cells overexpressing MERTK. Cells were transfected with an expression vector for MERTK for 24 h and then infected with EBOVΔVP30 at an MOI of 3.0 for 30 min. Cell lysates were immunoprecipitated with an anti-MERTK antibody and then immunoblotted. Data are representative of two independent experiments. IP, immunoprecipitation. WCE, whole-cell extract.
(TIF)
