# Supplementary figures and images for: HER2-mediated enhancement of Ebola virus entry
Source: PLoS Pathog. 2020 Oct 14;16(10):e1008900. doi: 10.1371/journal.ppat.1008900 (PMC7556532; doi:10.1371/journal.ppat.1008900)

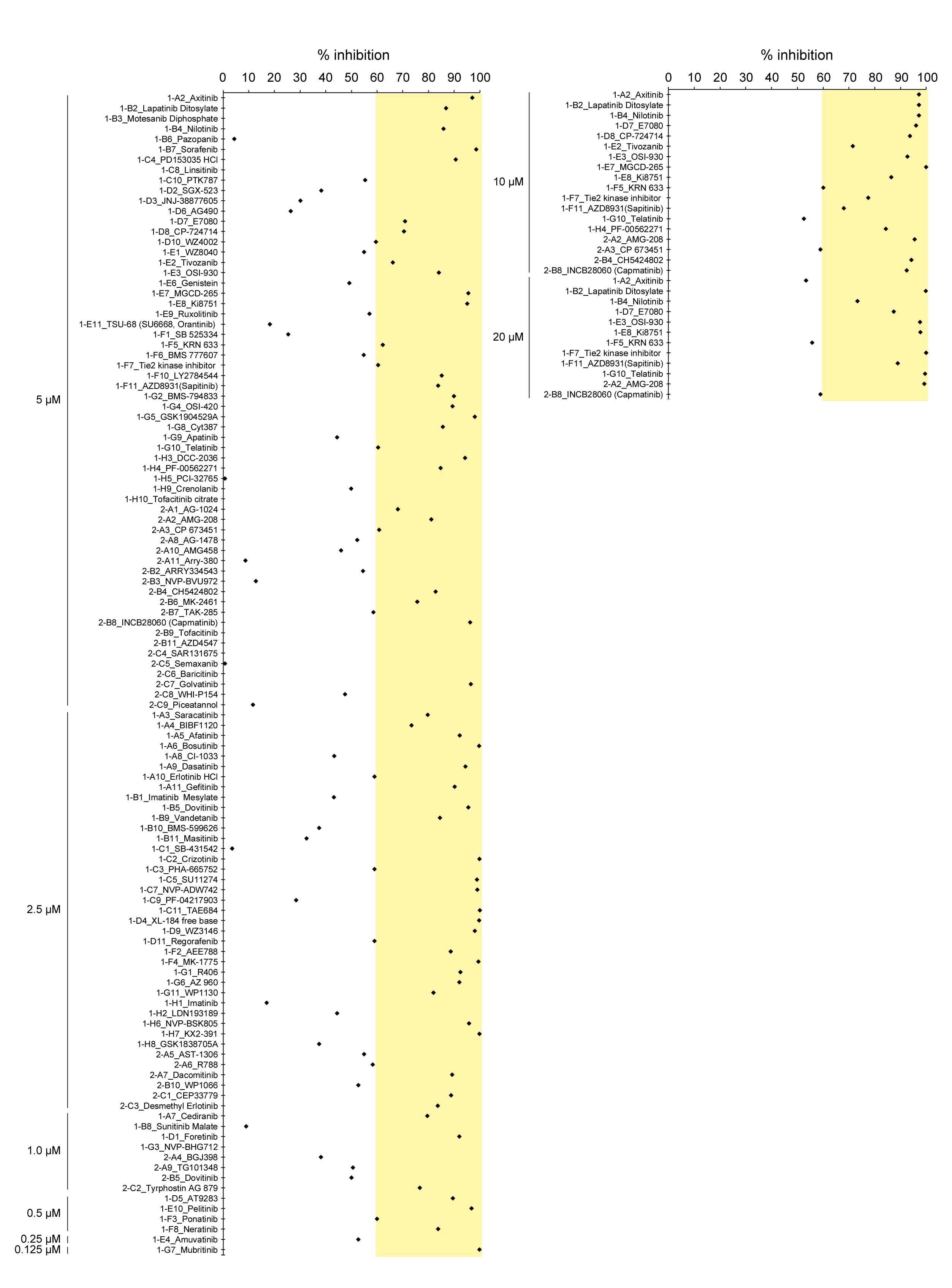

Supplement: S1 Fig — Dot plot of the inhibition of VSV-EBOV GP titers after treatment with RTK inhibitors. Vero cells were treated with each RTK inhibitor at the indicated concentration for 4 h prior to infection with VSV-EBOV GP at an MOI of 0.001. Virus titers were determined on day 2 post-infection and compared to those in control cells treated with 0.5% DMSO. Data are presented as means of at least two independent experiments. (TIF) [file ppat.1008900.s001.tif]

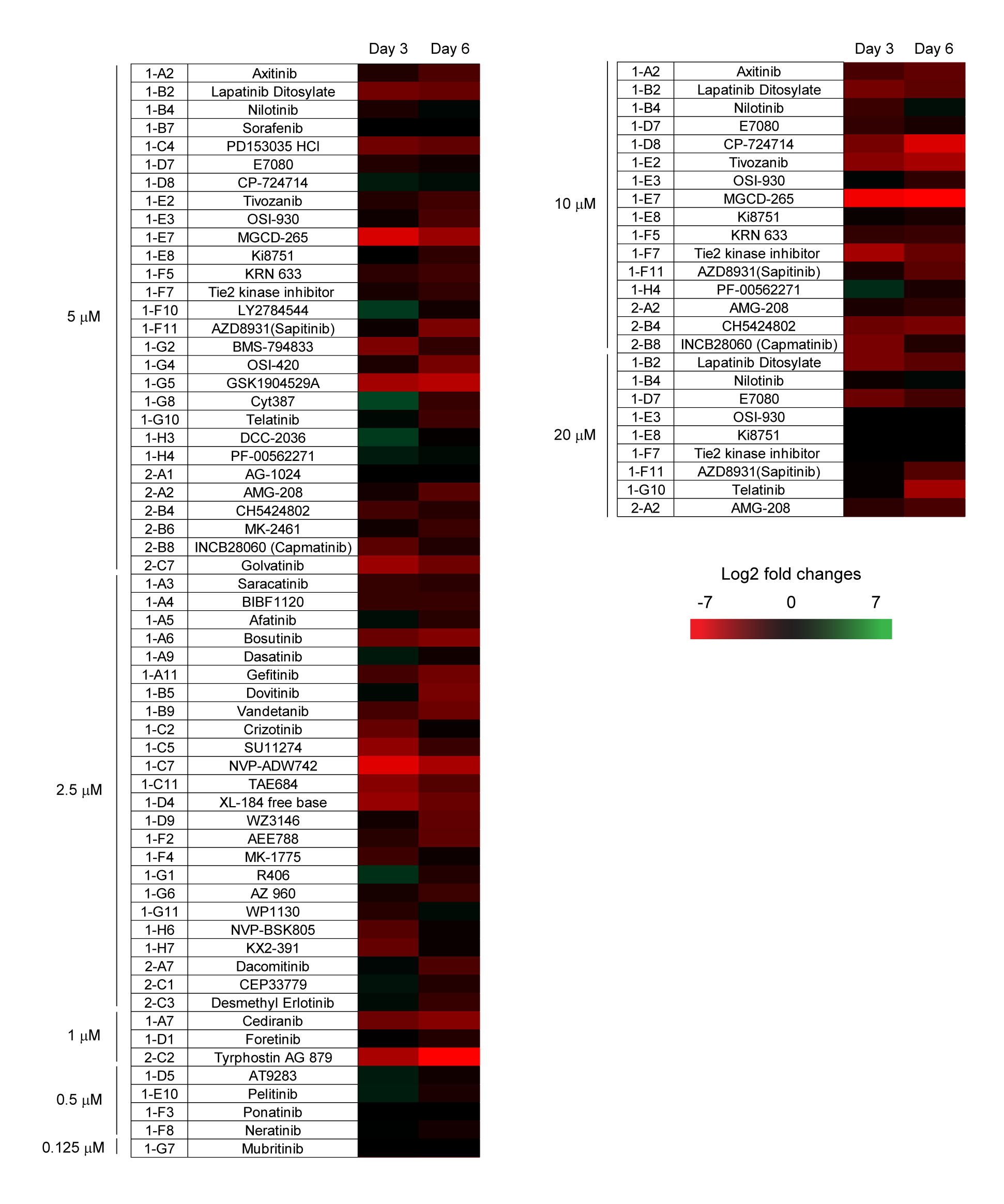

Supplement: S2 Fig — Heat map of fold changes in EBOVΔVP30 titers in the presence of RTK inhibitors. Vero VP30 cells were treated with each RTK inhibitor at the indicated concentration for 4 h prior to infection with EBOVΔVP30 at an MOI of 0.001. Virus titers were determined on days 3 and 6 post-infection and compared with those in the control cells treated with 0.5% DMSO. Data are presented as fold changes of means from at least three independent experiments. (TIF) [file ppat.1008900.s002.tif]

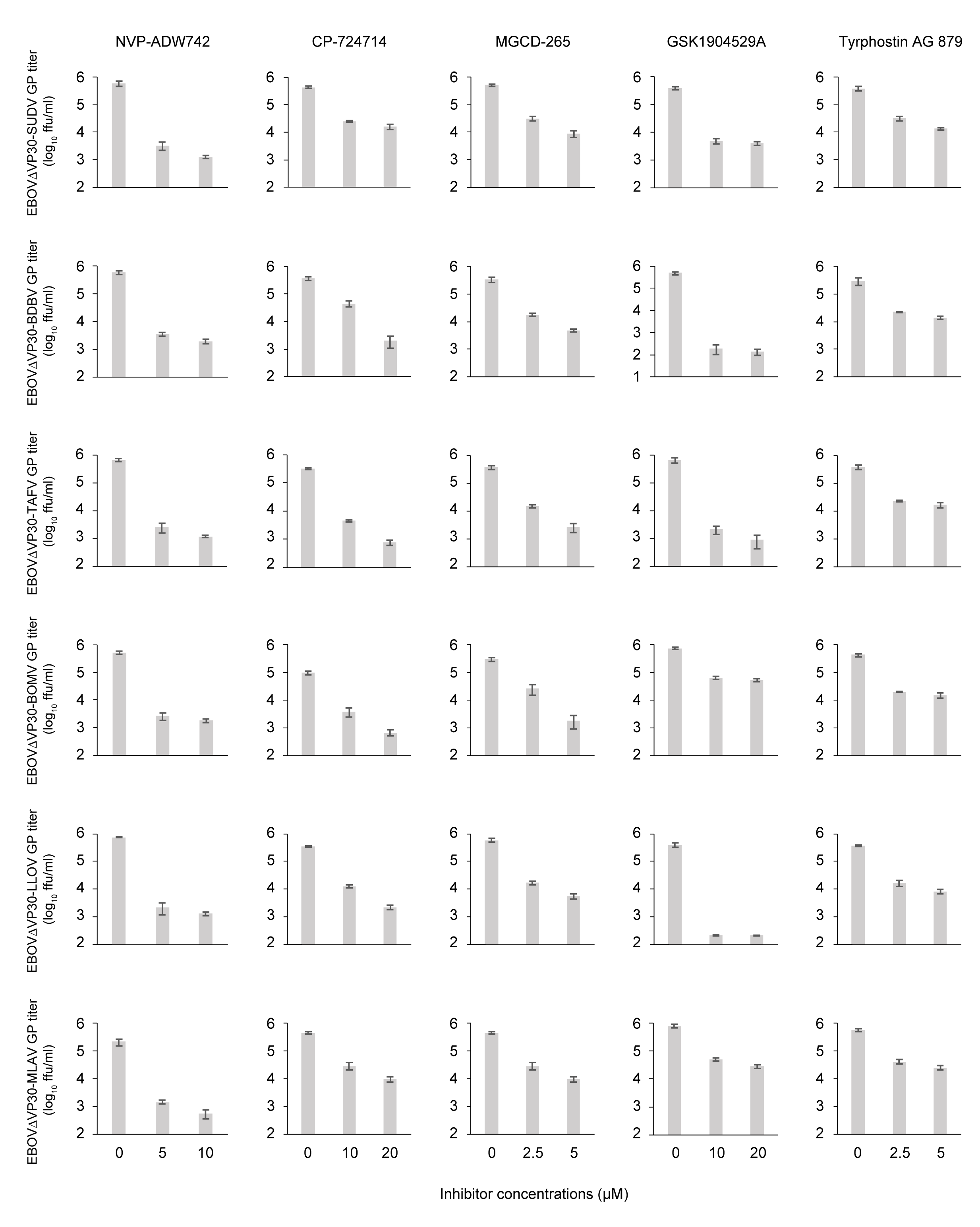

Supplement: S3 Fig — Titers of chimeric EBOVΔVP30 bearing the indicated filovirus GPs from infected Huh7.0 VP30 cells in the presence of RTK inhibitors. Cells were treated with each RTK inhibitor at the indicated concentration or with 0.5% DMSO for 4 h prior to infection with the viruses at an MOI of 0.01–0.002. Virus titers were determined on day 3 post-infection. Data are presented as means ± SD, and are representative of experiments performed in triplicate and repeated twice. SUDV, Sudan virus; BDBV, Bundibugyo virus; TAFV, Taï Forest virus; BOMV, Bombali virus; LLOV, Lloviu virus; MLAV, Měnglà virus. (TIF) [file ppat.1008900.s003.tif]

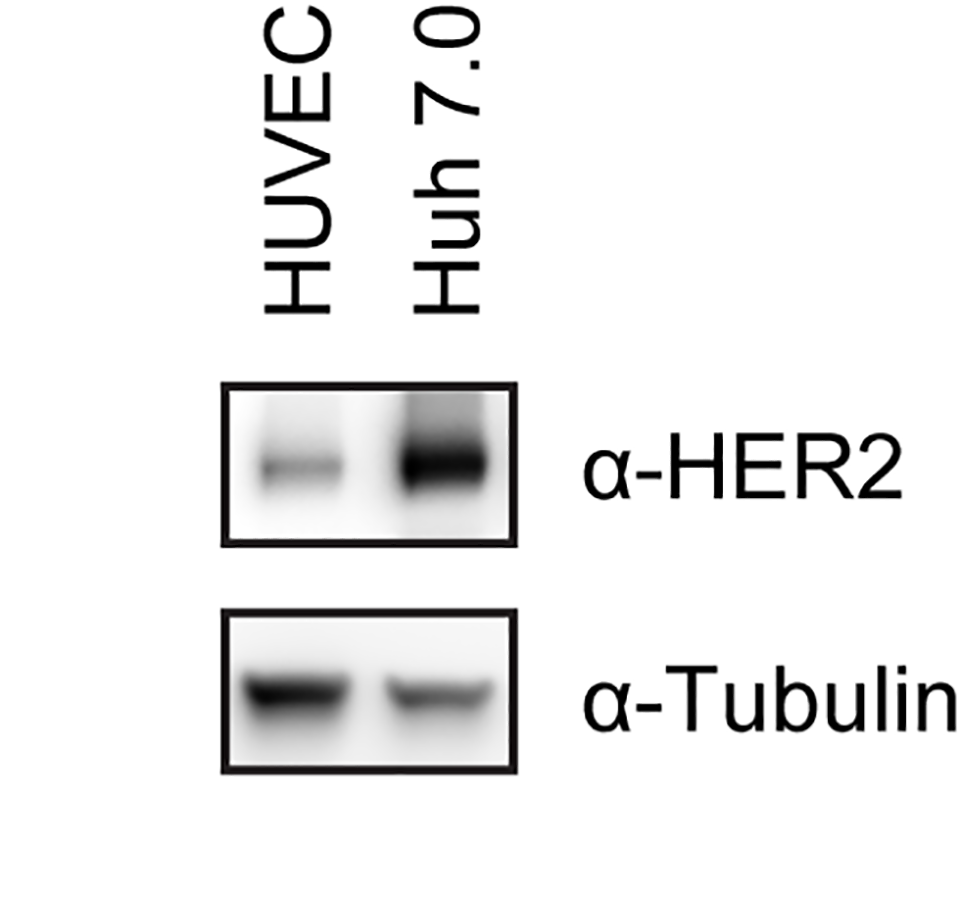

Supplement: S4 Fig — HER2 expression in HUVEC VP30 and Huh7.0 VP30 cells. The indicated protein expression levels were analyzed by immunoblotting. (TIF) [file ppat.1008900.s004.tif]

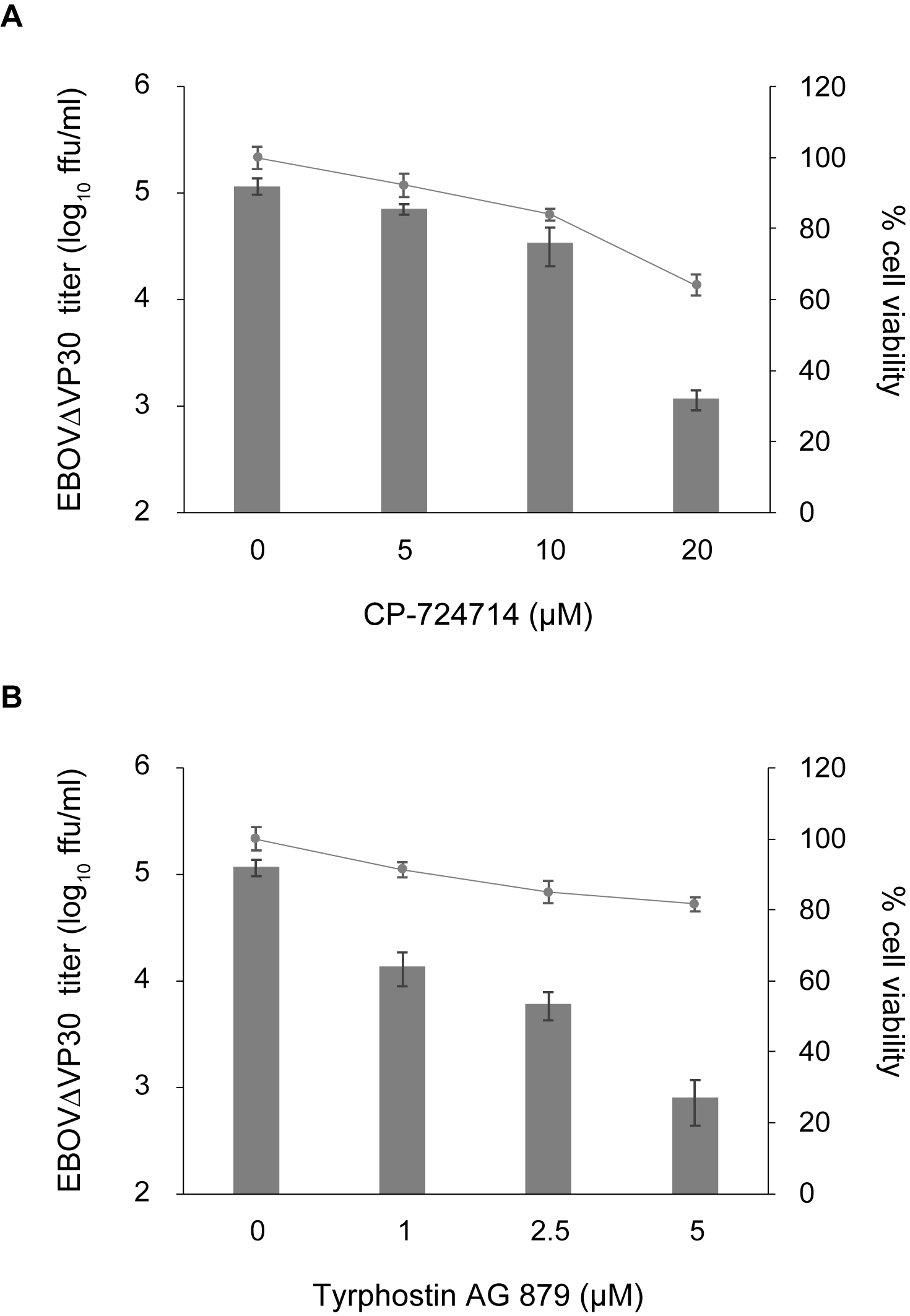

Supplement: S5 Fig — Titers of EBOVΔVP30-GFP (shown as bars) from HUVEC VP30 cells in the presence of the HER2 inhibitors CP-724714 (A) and Tyrphostin AG 879 (B). Cells were treated with increasing doses of the indicated inhibitors or with 0.5% DMSO for 4 h prior to infection with EBOVΔVP30 at an MOI of 0.005. Virus titers were determined on day 3 post-infection. In a separate set of experiments, cell viability (shown as continuous lines) after treatment with inhibitors for 3 days was measured by performing a cell viability assay. Data are presented as means ± SD, and are representative of experiments performed in triplicate and repeated twice. (TIF) [file ppat.1008900.s005.tif]

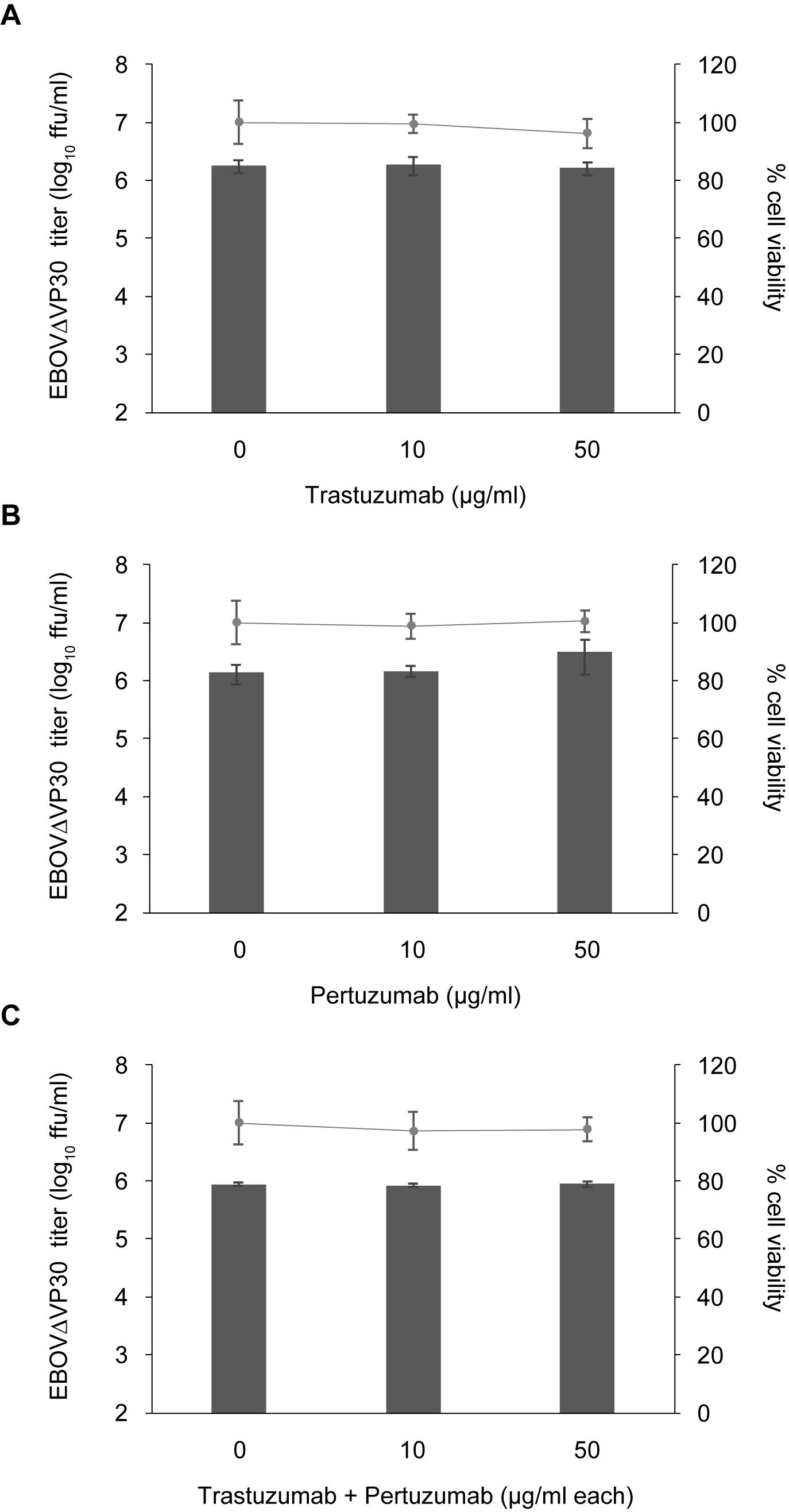

Supplement: S6 Fig — Titers of EBOVΔVP30-GFP (shown as bars) from Huh7.0 VP30 cells in the presence of the anti-HER2 antibodies Trastuzumab (A), Pertuzumab (B), and a combination of both (C). Cells were treated with the indicated concentrations of the antibodies for 1 h prior to infection with EBOVΔVP30 at an MOI of 0.01. Virus titers were determined on day 3 post-infection. In a separate set of experiments, cell viability (shown as continuous lines) after treatment with antibodies for 3 days was measured by performing a cell viability assay. Data are presented as means ± SD, and are representative of experiments performed in triplicate and repeated twice. (TIF) [file ppat.1008900.s006.tif]

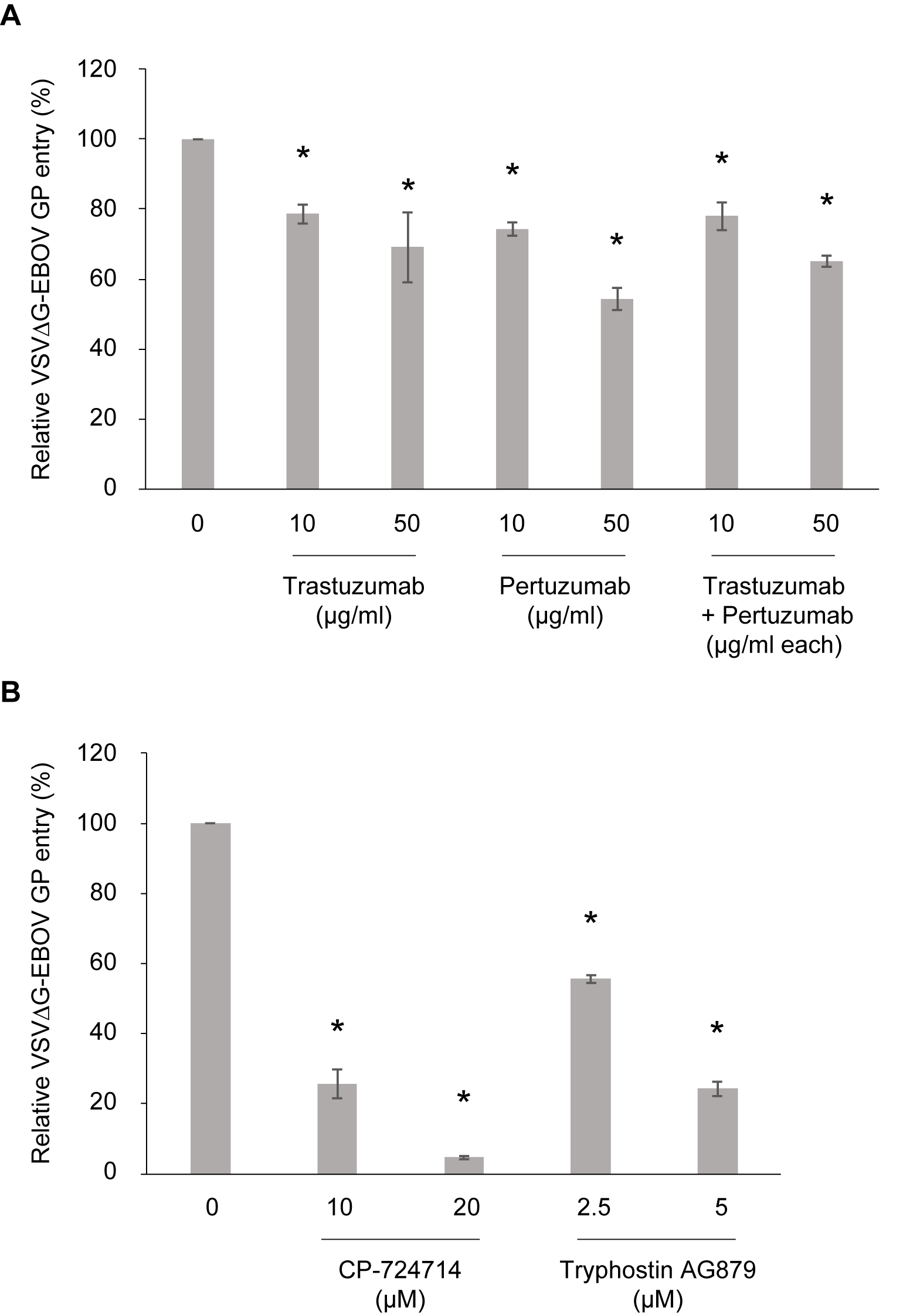

Supplement: S7 Fig — (A) Relative luciferase activity in Huh7.0 VP30 cells in the presence of the indicated anti-HER2 antibodies after infection with VSVΔG-EBOV GP virus at an MOI of 0.5. Data are presented as means ± SD of four independent experiments performed in triplicate. (*) indicates a statistically significant difference (p value ≤ 0.05) from the control. (B) Relative luciferase activity in Huh7.0 VP30 cells in the presence of the indicated HER2 inhibitors after infection with VSVΔG-EBOV GP virus at an MOI of 0.5. Data are presented as means ± SD of three independent experiments performed in triplicate. (*) indicates a statistically significant difference (p value ≤ 0.05) from the control. (TIF) [file ppat.1008900.s007.tif]

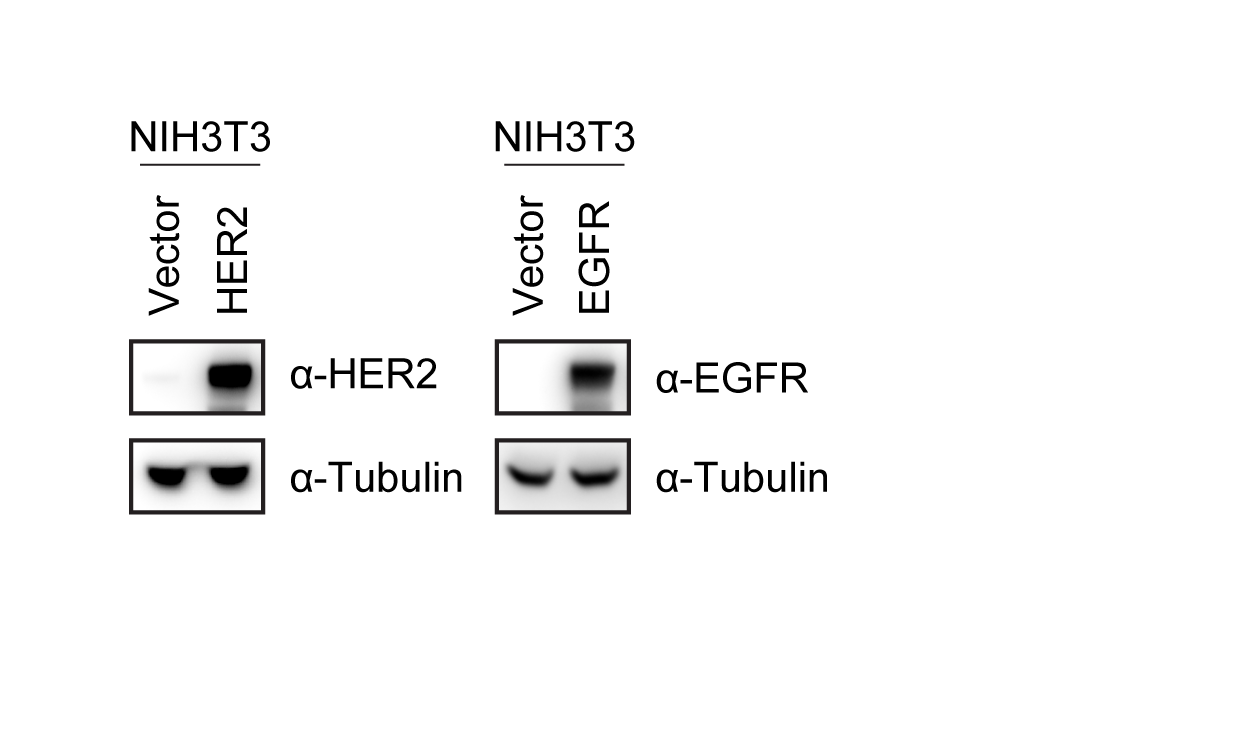

Supplement: S8 Fig — HER2 and EGFR expression in NIH3T3 stable cell lines expressing either HER2 or EGFR. The indicated protein expression levels were analyzed by immunoblotting. (TIF) [file ppat.1008900.s008.tif]

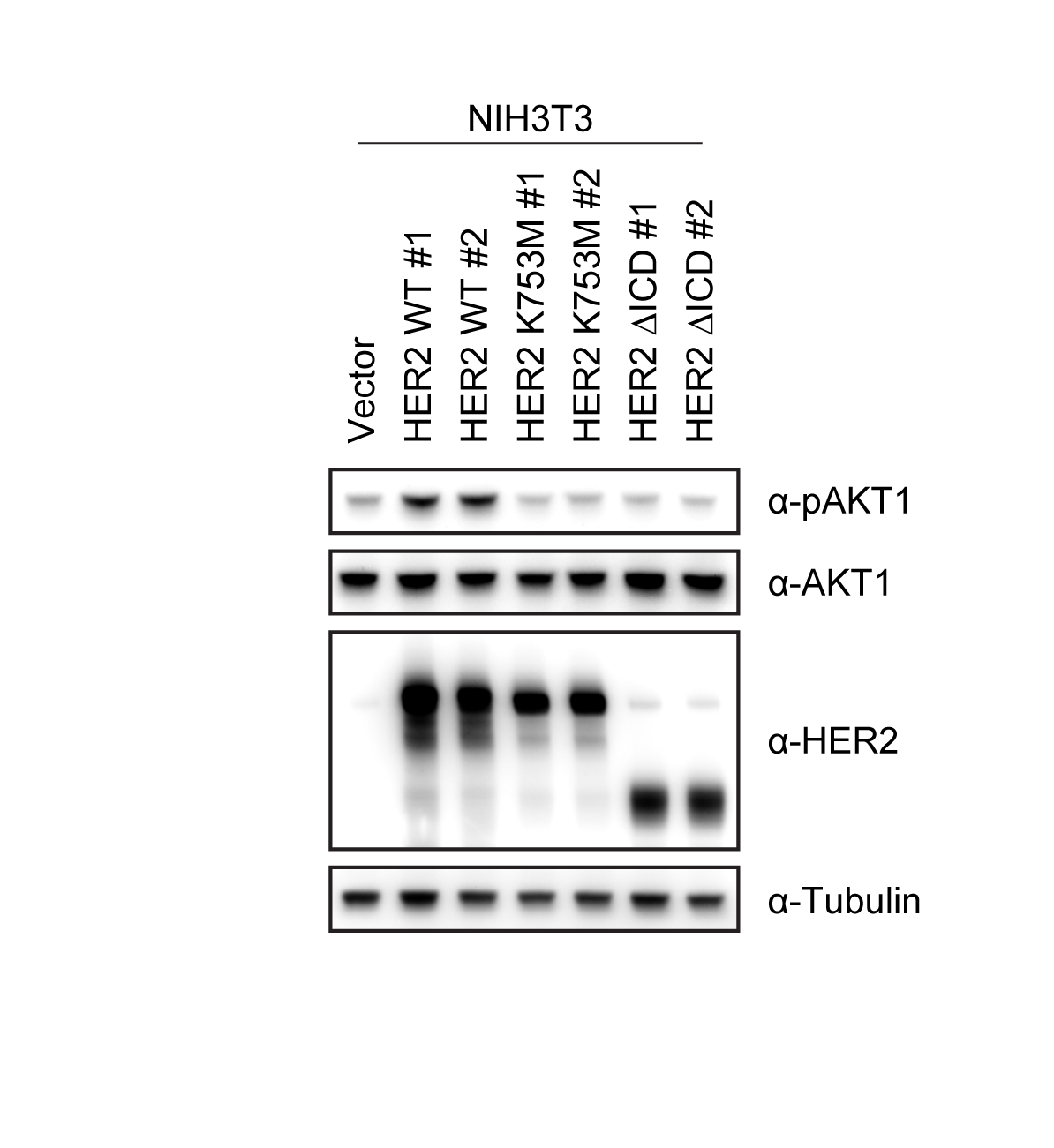

Supplement: S9 Fig — Phosphorylated AKT1 in NIH3T3 stable cell lines expressing either HER2 WT or the indicated kinase-deficient mutants or in an empty vector control cell line. The indicated protein expression levels were analyzed by immunoblotting. The numbers indicate two different stable cell line populations generated in the same setting. (TIF) [file ppat.1008900.s009.tif]

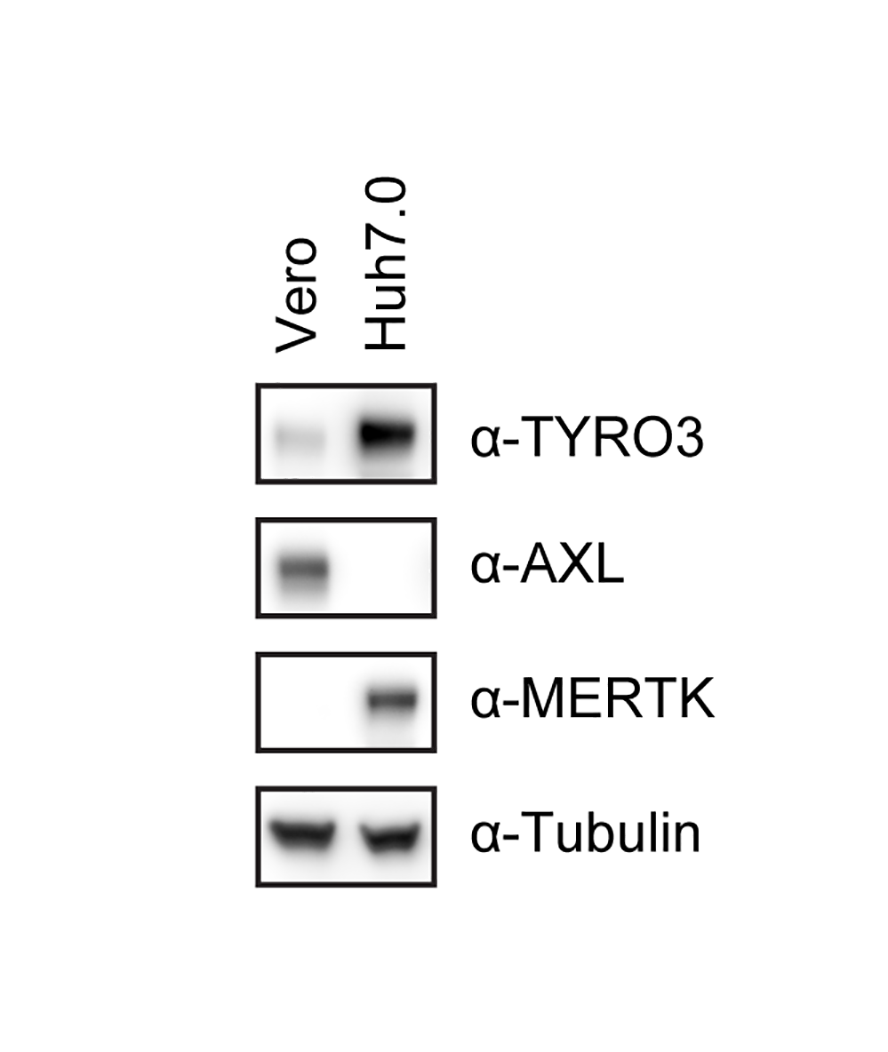

Supplement: S10 Fig — Expression of TYRO3, AXL, and MERTK in Vero and Huh7.0 cells. The indicated protein expression levels were analyzed by immunoblotting. (TIF) [file ppat.1008900.s010.tif]

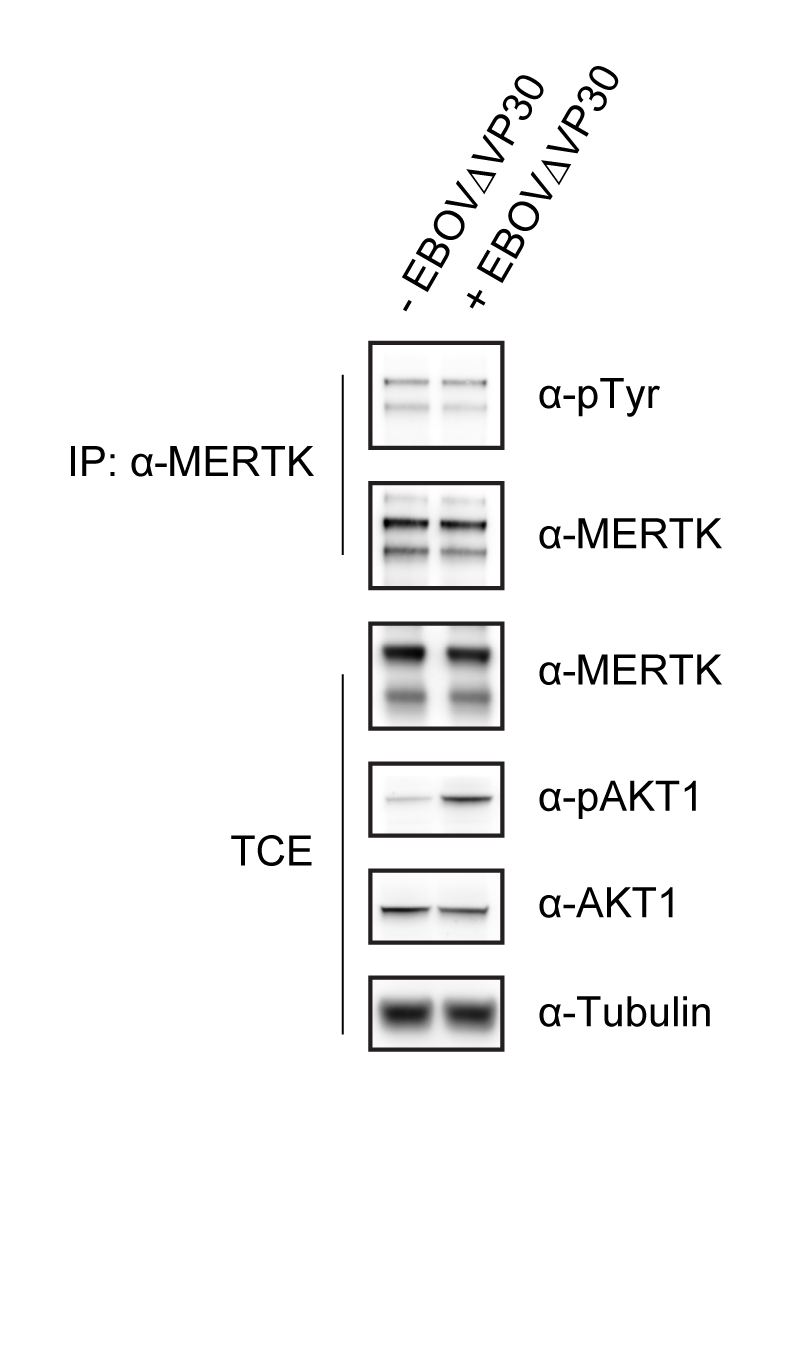

Supplement: S11 Fig — The phosphorylation level of MERTK in Huh7.0 cells overexpressing MERTK. Cells were transfected with an expression vector for MERTK for 24 h and then infected with EBOVΔVP30 at an MOI of 3.0 for 30 min. Cell lysates were immunoprecipitated with an anti-MERTK antibody and then immunoblotted. Data are representative of two independent experiments. IP, immunoprecipitation. WCE, whole-cell extract. (TIF) [file ppat.1008900.s011.tif]
